# Comparative Genome Analysis of *Lactococcus lactis* Indicates Niche Adaptation and Resolves Genotype/Phenotype Disparity

**DOI:** 10.3389/fmicb.2019.00004

**Published:** 2019-01-31

**Authors:** Michiel Wels, Roland Siezen, Sacha van Hijum, William J. Kelly, Herwig Bachmann

**Affiliations:** ^1^NIZO Food Research B.V., Ede, Netherlands; ^2^TI Food and Nutrition, Wageningen, Netherlands; ^3^Centre for Molecular and Biomolecular Informatics, Radboud Institute for Molecular Life Sciences, Radboud University Medical Center, Nijmegen, Netherlands; ^4^Microbial Bioinformatics, Ede, Netherlands; ^5^AgResearch Ltd, Palmerston North, New Zealand; ^6^Systems Bioinformatics, Vrije Universiteit Amsterdam, Amsterdam, Netherlands

**Keywords:** *Lactococcus lactis*, comparative genomics, gene-trait matching, niche adaptation, dairy fermentation

## Abstract

*Lactococcus lactis* is one of the most important micro-organisms in the dairy industry for the fermentation of cheese and buttermilk. Besides the conversion of lactose to lactate it is responsible for product properties such as flavor and texture, which are determined by volatile metabolites, proteolytic activity and exopolysaccharide production. While the species *Lactococcus lactis* consists of the two subspecies *lactis* and *cremoris* their taxonomic position is confused by a group of strains that, despite of a *cremoris* genotype, display a *lactis* phenotype. Here we compared and analyzed the (draft) genomes of 43 *L. lactis* strains, of which 19 are of dairy and 24 are of non-dairy origin. Machine-learning algorithms facilitated the identification of orthologous groups of protein sequences (OGs) that are predictors for either the taxonomic position or the source of isolation. This allowed the unambiguous categorization of the genotype/phenotype disparity of ssp. *lactis* and ssp. *cremoris* strains. A detailed analysis of phenotypic properties including plasmid-encoded genes indicates evolutionary changes during niche adaptations. The results are consistent with the hypothesis that dairy isolates evolved from plant isolates. The analysis further suggests that genomes of *cremoris* phenotype strains are so eroded that they are restricted to a dairy environment. Overall the genome comparison of a diverse set of strains allowed the identification of niche and subspecies specific genes. This explains evolutionary relationships and will aid the identification and selection of industrial starter cultures.

## Introduction

*Lactococcus lactis* is a non-motile, non-spore-forming, low G+C, Gram-positive lactic acid bacterium. It is used as a starter culture in the production of a wide range of fermented dairy products where it contributes to food preservation, flavor and texture formation (Smid and Kleerebezem, 2014). *Lactococcus lactis* has been isolated from a variety of environments where its primary role is the initiation of fermentation by the rapid utilization of available carbohydrates to produce lactic acid. Wild-type plant- and animal-associated strains are able to ferment a wide range of mono-, di- and tri-saccharides. Members of this species have also acquired the ability to ferment lactose with some strains rapidly transitioning to become specialists adapted to the homogeneous milk environment. The industrial importance of *L. lactis* is demonstrated by a global cheese production of close to 2 × 10^7^ tons in 2015 (Bulletin of the International Dairy Federation 2016), and based on that we estimate that over 10^20^ lactococci are being consumed by humans annually.

The taxonomy of *L. lactis* is currently phenotypically based (Schleifer et al., 1985; van Hylckama Vlieg et al., 2006; Rademaker et al., 2007) with two main subspecies (*lactis* and *cremoris*) and one biovar (*lactis* biovar *diacetylactis*). On the basis of their 16S rRNA gene sequence, these two subspecies are estimated to have diverged some 17 million years ago (Bolotin et al., 2004). Several studies have shown that two genotypic lineages (also called subspecies *cremoris* and *lactis*) exist, but that strains with the ssp. *cremoris* genotype can show either phenotype making accurate identification confusing (van Hylckama Vlieg et al., 2006; Kelly et al., 2010; Cavanagh et al., 2015). Attempts to explain this disparity have been made by detailed analysis of selected genes (Godon et al., 1992; Beimfohr et al., 1997; Pu et al., 2002) and comparative genomics (Kelleher et al., 2017), but to date the genes underlying this disparity are unknown. Although both subspecies are used as starters by the dairy industry, defined strains with the ssp. *cremoris* phenotype are preferred for Cheddar cheese production because of their desirable properties especially in relation to acid production, flavor development and bacteriophage resistance (Limsowtin et al., 1996). Two additional phenotypic subspecies (*hordniae* and *tructae*) have been described (Latorre Guzman et al., 1977; Perez et al., 2011), although at the genome level these cluster within subspecies *lactis* and *cremoris*, respectively.

Strains with the *lactis* phenotype show high genetic diversity (Rademaker et al., 2007; Passerini et al., 2010) and have been isolated from a variety of sources, most commonly fresh or fermenting plant material or milk and dairy environments. The *cremoris* phenotype is only found in dairy environments and many attempts to isolate similar cultures from environmental sources have not been successful (Klijn et al., 1995; Salama et al., 1995). These *cremoris* phenotype strains cluster closely together (Rademaker et al., 2007) and are likely to have a common origin. There is a considerable body of evidence from comparative and experimental studies that suggests that dairy isolates have evolved from plant isolates (van Hylckama Vlieg et al., 2006; Kelly et al., 2010; Siezen et al., 2010b; Bachmann et al., 2012; Kelleher et al., 2017), and many of the properties that permit rapid growth in milk (lactose utilization, proteolysis, peptide transport) are located on plasmids (Ainsworth et al., 2014b). As many as 12 plasmids (van Mastrigt et al., 2018) and plasmid sizes up to 140 kb have been reported in *L. lactis* (Kojic et al., 2005). Numerous industrially important traits (bacteriophage resistance, bacteriocin production, exopolysaccharide production) are also frequently plasmid located.

In a previous study comparative genome hybridization was used to assess the gene content of 38 lactococci and the data was matched with 207 phenotypes, which gave insight into niche-specific differences (Siezen et al., 2011). The limitation of comparative genome hybridization is that only genes present on a multi-strain DNA microarray can be detected while new genes can not be detected. This can only be overcome by using genome sequencing data. The draft genomes of the *L. lactis* strains used by Siezen et al. (2011) were recently sequenced (Backus et al., 2017; Wels et al., 2017) and here we analyzed and compared these strains plus several publicly available fully sequenced *L. lactis* genomes. We used machine-learning algorithms (Bayjanov et al., 2013) to identify orthologous groups of protein sequences (OGs) that are predictors for either the taxonomic position or the source of isolation. Genome comparison provides evidence of the genetic events that have shaped the evolution of the *L. lactis* and explains the current genotype/phenotype disparity of ssp *lactis* and ssp *cremoris*.

## Materials and Methods

### Strains

All genome sequences of the strains used in this study (Table 1) were obtained from the public databases and re-annotated with the same annotation pipeline (Seemann, 2014) to prevent gene calling and function annotation differences between genomes caused by the use of different gene calling software or annotation pipelines.

**Table 1:** Strains used in this study.

Strain	Subspecies (genotype)	Phenotype	Origin	Genome size (Mb)	Proteins	Plasmid content (Kb)	Complete/draft	Accession #
KW10	*L. lactis* ssp. c*remoris*	Lactis	Kaanga Wai	2.36	2285	0	Draft	PRJNA286840
KW2	*L. lactis* ssp. c*remoris*	Lactis	Kaanga Wai	2.43	2327	0	Complete	PRJNA189982
V4	*L. lactis* ssp. c*remoris*	Lactis	Raw sheep milk	2.55	2591	67.78	Draft	PRJNA286840
N41	*L. lactis* ssp. c*remoris*	Lactis	Soil and grass	2.61	2655	154.58	Draft	PRJNA286840
NCDO763	*L. lactis* ssp. c*remoris*	Lactis	Dairy starter	2.48	2553	100.62	Draft	PRJNA286840
MG1363	*L. lactis* ssp. c*remoris*	Lactis	Dairy starter	2.53	2647	0	Complete	PRJNA18797
SK110	*L. lactis* ssp. c*remoris*	Cremoris	Dairy Starter	2.46	2597	149	Draft	PRJNA286840
AM2	*L. lactis* ssp. c*remoris*	Cremoris	Dairy starter	2.47	2617	59.04	Draft	PRJNA286840
SK11	*L. lactis* ssp. c*remoris*	Cremoris	Dairy starter	2.44	2780	150	Complete	PRJNA401
A76	*L. lactis* ssp. c*remoris*	Cremoris	Cheese factory	2.45	2786	120	Complete	PRJNA74685
UC509_9	*L. lactis* ssp. c*remoris*	Cremoris	Dairy starter	2.25	2600	210	Complete	PRJNA76597
B40	*L. lactis* ssp. c*remoris*	Cremoris	Dairy starter	2.48	2641	153.47	Draft	PRJNA286840
FG2	*L. lactis* ssp. c*remoris*	Cremoris	Dairy starter	2.57	2729	138.97	Draft	PRJNA286840
HP	*L. lactis* ssp. c*remoris*	Cremoris	Dairy starter	2.38	2556	126.1	Draft	PRJNA286840
LMG6897	*L. lactis* ssp. c*remoris*	Cremoris	Cheese starter	2.36	2527	100.11	Draft	PRJNA286840
LMG8526	*L. lactis* ssp. *lactis*	Lactis	Chinese radish seeds	2.48	2459	75.36	Draft	PRJNA294255
KF282	*L. lactis* ssp. *lactis*	Lactis	Mustard and cress	2.65	2610	16.6	Draft	PRJNA294255
LMG8520	*L. lactis* ssp. *Hordniae*	Lactis	Leaf hopper	2.43	2683	126.93	Draft	PRJNA294255
IO_1	*L. lactis* ssp. *lactis*	Lactis	Drain water	2.42	2333	0	Complete	PRJDA68077
K231	*L. lactis* ssp. *lactis*	Lactis	White Kimchi	2.34	2311	17.17	Draft	PRJNA294255
Li-1	*L. lactis* ssp. *lactis*	Lactis	Grass	2.48	2450	90.16	Draft	PRJNA294255
KF24	*L. lactis* ssp. *lactis*	Lactis	Alfalfa sprouts	2.61	2670	51.05	Draft	PRJNA294255
LMG9447	*L. lactis* ssp. *lactis*	Lactis	Frozen peas	2.69	2792	114.17	Draft	PRJNA294255
ATCC19435	*L. lactis* ssp. *lactis*	Lactis	Dairy starter	2.54	2581	64.11	Draft	PRJNA294255
E34	*L. lactis* ssp. *lactis*	Lactis	Silage	2.38	2310	0.8	Draft	PRJNA294255
K337	*L. lactis* ssp. *lactis*	Lactis	White Kimchi	2.42	2407	0	Draft	PRJNA294255
KF201	*L. lactis* ssp. *lactis*	Lactis	Sliced mixed vegetables	2.38	2344	0	Draft	PRJNA294255
M20	*L. lactis* ssp. l*actis*	Diacetylactis	Soil	2.67	2681	41.86	Draft	PRJNA294255
N42	*L. lactis* ssp. *lactis*	Lactis	Soil and grass	2.73	2769	165.55	Draft	PRJNA294255
DRA4	*L. lactis* ssp. l*actis*	Diacetylactis	Dairy starter	2.44	2503	0	Draft	PRJNA294255
IL1403	*L. lactis* ssp. l*actis*	Lactis	Dairy starter	2.37	2448	0	Complete	PRJNA503975
CV56	*L. lactis* ssp. *lactis*	Lactis	Human vagina	2.4	2563	120	Complete	PRJNA60377
ML8	*L. lactis* ssp. *lactis*	Lactis	dairy starter	2.5	2576	142.37	Draft	PRJNA294255
UC317	*L. lactis* ssp. *lactis*	Lactis	Dairy starter	2.49	2565	0	Draft	PRJNA294255
LMG14418	*L. lactis* ssp. *lactis*	Lactis	Bovine milk	2.41	2445	19.01	Draft	PRJNA294255
KLDS 4.0325	*L. lactis* ssp. *lactis*	Lactis	Koumiss	2.59	2656	170	Complete	PRJNA218564
KF147	*L. lactis* ssp. *lactis*	Lactis	Mung bean sprouts	2.6	2638	40	Complete	PRJNA294255
KF7	*L. lactis* ssp. *lactis*	Lactis	Alfalfa sprouts	2.37	2357	20.96	Draft	PRJNA294255
KF67	*L. lactis* ssp. *lactis*	Lactis	Grapefruit juice	2.68	2669	49.11	Draft	PRJNA294255
KF196	*L. lactis* ssp. *lactis*	Lactis	Japanese Kaiware shoots	2.45	2387	26.24	Draft	PRJNA294255
KF146	*L. lactis* ssp. *lactis*	Lactis	Alfalfa and radish sprouts	2.58	2546	59.05	Draft	PRJNA294255
KF134	*L. lactis* ssp. *lactis*	Lactis	Alfalfa and radish sprouts	2.47	2418	1.1	Draft	PRJNA294255
LMG9446	*L. lactis* ssp. *lactis*	Lactis	Frozen peas	2.49	2494	77.32	Draft	PRJNA294255
P7266	*Lactococcus* sp.		Litter on pasture	2	1980	24.72	Draft	PRJNA286840

### Genome Comparison

All protein sequences encoded in the genomes were compared using OrthoMCL (Li et al., 2003). OrthoMCL uses all-against-all protein BLAST and subsequently performs MCL clustering, grouping proteins with high sequence similarity. In this way orthologs (genes in different species that evolved from a common ancestral gene by speciation) are predicted.

The OrthoMCL output matrix containing OGs, i.e., gene families, was used as an input to generate a database (in MS Excel) in which the information about the location (contig-level coordinates) and length of orthologous proteins of all *L. lactis* genomes were aligned. Multiple sequence alignment (MSA) files were made where the nucleotide and protein sequences within OGs were aligned, to facilitate identification of pseudogenes (encoding incomplete proteins), and to identify correct start codons.

Detailed examination of specific genome locations and functional analysis was performed within the Integrated Microbial Genome platform (IMG-ER^1^) (Chen et al., 2017).

### Core and Pan Genome Determination

All OGs with a single gene copy in each of the strains were selected from the OrthoMCL output. The nucleotide sequences of these OGs were aligned using Muscle (Edgar, 2004) and alignment positions with nucleotide differences were isolated and stored in a single artificial sequence. This sequence was used as a basis to generate a whole-genome-based phylogeny using FastTree (Price et al., 2009). Re-rooting of the tree was performed using one of the two subspecies (e.g., ssp. *cremoris*) as an outgroup. The pangenome was scored by counting all OGs within the complete set of studied genomes. Core and pan genome sizes were calculated on defining the average increase or decrease of the pan/core genome, including the standard variation.

### Subspecies, Niche and Phenotype Matching With the Genotype

For the matching of the phenotypes, described in (Bayjanov et al., 2013), as well as the identification of genes associated with subspecies and niche, we used the Phenolink scripts (Bayjanov et al., 2012), running on a local Linux operating system. For the phenotype matching, pseudogenes were regarded as an intermediate state between presence and absence. In this way, the pseudogene could be regarded as both present as well as absent in the genome. Heatmap figures were prepared with the OGs identified by genotype-phenotype matching as most discriminating for either the subspecies or the isolation source, or for plasmid-derived OGs. Data visualization was done using R^2^. The heatmap function using Manhattan or Euclidean distance matrices and complete hierarchical clustering and data scaling was used for the generation of heatmaps.

### Plasmid Analysis

For plasmid isolation, strains were individually cultured in GM17 medium and pools of equal volumes of fully grown cultures were prepared. Plasmid DNA isolation was performed by an alkaline lysis method as described previously (Vos et al., 1989) followed by phenol chloroform extraction and DNA precipitation (Sambrook and Russell, 2001). The plasmid sequencing was performed at BaseClear B.V. (Leiden, Netherlands) with 100-bp paired-end libraries on an Illumina HiSeq 2000. The sequence reads that resulted from the dedicated plasmid DNA isolation were used to map against the contigs of the genome sequences using breseq (Deatherage and Barrick, 2014). An increased contig coverage was used as the main criterion to determine if contigs were of plasmid origin or not. Manual inspection of these contigs was performed to remove chromosomal repeat elements such as rRNA clusters, transposable elements and phages.

## Results

### Core and Pangenome

We compared the genomes of 44 *L. lactis* strains (Table 1), 36 of which featured in a previous study using comparative genome hybridization and extensive phenotyping (Siezen et al., 2011). The strains were mainly isolated from either a dairy environment or plant material with the exception of one isolate each from human, leafhopper, soil and sink drain water. The draft genomes of 34 strains were recently described (Backus et al., 2017; Wels et al., 2017) while complete genome sequences are available for the other strains.

**FIGURE 1:**
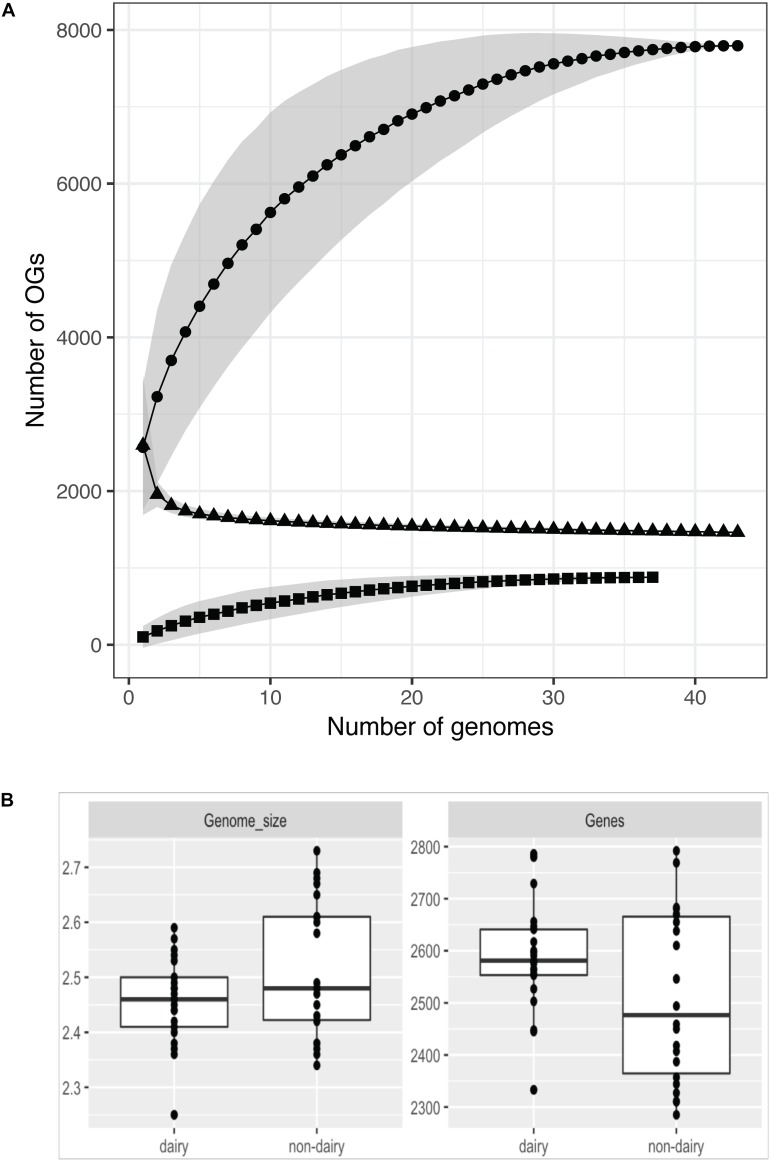
Pangenome (circles), core-genome (triangles), and plasmid pangenome (squares) assessment of 43 strains **(A)**. The number of OGs increases with the number of strains sequenced for the chromosomal and plasmid DNA while the core genome size decreases. For all three the numbers level off above 20–30 sequenced genomes. Variation of the strains chosen for the analysis results in a different number of identified OGs (given as gray areas). Not all analyzed strains harbor plasmids which can be seen in the plasmid pangenome data. Genome size and number of genes (including pseudogenes) per strains in dairy and non-dairy isolates **(B)**.

The average genome size of 43 strains (excluding strain P7266 – see reasoning later) is 2.49 Mb with an average 2548 protein coding sequences (Figure 1). The largest draft genome sequence is that of *L. lactis* ssp. *lactis* N42 with a genome size of 2.73 Mb while the smallest is *L. lactis* ssp. *lactis* K231 at 2.34 Mb. For strains with complete genome sequences the largest chromosome is from strain *L. lactis* ssp. *lactis* KF147 (2.60 Mb) and the smallest from *L. lactis* ssp. *cremoris* UC509.9 (2.25 Mb). By comparing the 43 *L. lactis* genomes using OrthoMCL 7795 orthologous groups (OGs) were identified, each corresponding to a different OG (Supplementary Table 1/Sheet 1). This set of OGs is often referred to as the pangenome and is considered to be the full complement of genes that can be found in a species. The size of the pangenome of the 43 *L. lactis* strains levels off at around 7800 OGs and is larger than that found in other LAB (Frese et al., 2011; Siezen and van Hylckama Vlieg, 2011; Dutilh et al., 2013; Smokvina et al., 2013), especially when corrected for genome size. Of the pangenome OGs, about 11% (879) are predicted to be of plasmid origin (see below) which is higher than observed in other species such as *Lactobacillus paracasei* (Smokvina et al., 2013), where this fraction is ∼5%.

Of the 7795 identified OGs, 1463 were found to be conserved among the 43 strains (Supplementary Table 1/Sheet 1). Of these 1463 genes, 70 were identified as being unique to the *L. lactis* species based on (i) genes in the LaCOG prediction (Makarova and Koonin, 2007) and (ii) conserved among all *L. lactis* genomes analyzed. Among this set of 70 OGs 46 were annotated as “hypothetical protein” (Supplementary Table 1/Sheet 2). The remainder encoded, amongst others functions, a L-lactate dehydrogenase, a cation transporter, other transporters with unknown specificity, an NADH oxidase, several stress proteins and a late competence protein.

### Evolution and Niche Adaptation

**FIGURE 2:**
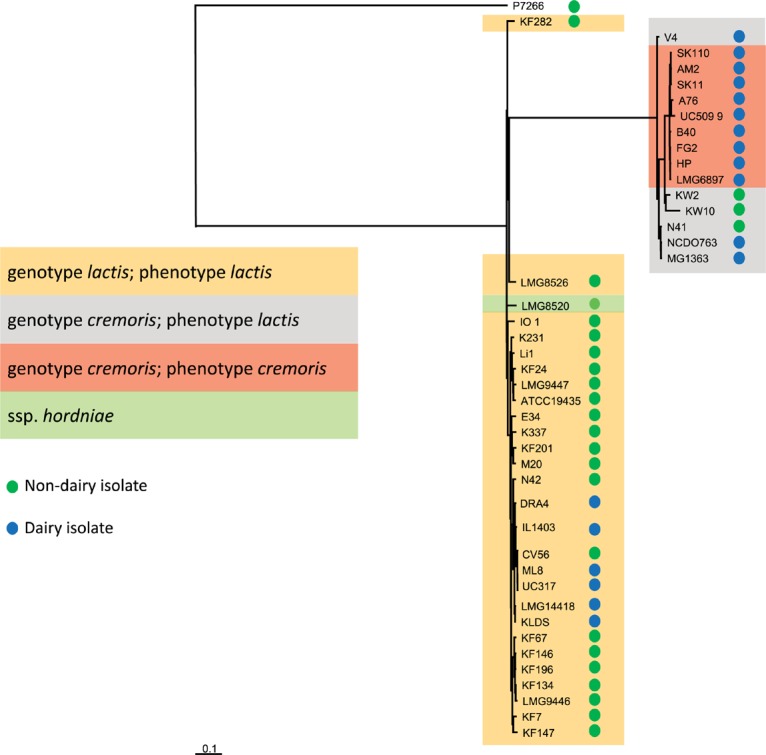
Phylogenetic distance was measured by single nucleotide differences between different strains within the core genome. Trees were inferred based on approximate maximum-likelihood. P7266 was used as an outgroup in the tree construction based on having the largest distance to the other strains.

Nucleotide variation in the core genes of all 44 strains (including strain P7266), which we defined as OGs occurring with 1 copy per genome, was used to determine the evolutionary relatedness of the strains (Figure 2 and Supplementary Figure 1). The tree was constructed in the same manner as described previously (Smokvina et al., 2013) with the difference that nucleotide sequences were used for alignments of the OGs to obtain a more detailed view of the relationship between genome difference and evolutionary time. Figure 2 shows strain P7266 forms an outgroup. Manual inspection of the 16S rRNA gene of this strain showed that it is not a *L. lactis*, but probably a member of another *Lactococcus* species, closely related to *L. taiwanensis* (data not shown). This strain was therefore was not included in subsequent analysis.

Figure 2 clearly shows the separation of strains into the ssp. *lactis* and *cremoris* clades which is consistent with earlier genotypic analysis of *L. lactis* (Rademaker et al., 2007). However, the clear identification of a *cremoris* clade that overlaps with a ssp. *lactis* phenotype was not possible with previous genotypic analyses that were either based on small subunit rRNA or on a five-locus Multi Locus Sequence Analysis (MLSA) (Rademaker et al., 2007). The analysis of the 43 genomes suggests that the *cremoris* genotype/*cremoris* phenotype strains share a common ancestor with the *cremoris*/*lactis* variants and that the *cremoris*/*cremoris* strains only evolved once (Figure 2). When focusing on the source of isolation, with the exception of KW2, KW10, and N41, all subspecies *cremoris* strains used in this study are isolated from a dairy environment, while the distribution of isolation source among the subspecies *lactis* strains is more spread over the dairy and non-dairy niches (Figure 2). The dairy isolated ssp. *lactis* strains cluster together in a clear branch of this subspecies (Figure 2 from N42 to KLDS). The only *L. lactis* ssp. *hordniae* strain (LMG8520) clusters closely to the ssp. *lactis* (Figure 2). The identified *L. lactis* ssp. *lactis* biovar *diacetylactis* strains also cluster within subspecies *lactis* clade.

While the draft genomes used here do not allow a precise analysis of the number of pseudogenes, an analysis of OGs that occur as pseudogenes in 8 or more strains shows that many more pseudogenes occur in the *cremoris/cremoris* clade (Supplementary Table 2). Consequently, the number of functional genes might be lower in these dairy isolates. The *cremoris* phenotype strains contain many identical pseudogenes resulting from adaptation to the dairy environment and indicative of their common ancestry.

Most of the differences above are consistent with the hypothesis that the dairy isolates of *L. lactis* evolved from plant isolates. This is most clearly shown by the genome degradation that has occurred in the dairy ssp *cremoris* strains as illustrated by examination of the complement of chromosomal genes that encode glycoside hydrolases (GHs) involved in carbohydrate metabolism. While the wildtype strains have >45 GHs, in the dairy strains many of these have been lost or are now pseudogenes, with HP and FG2 having only 19 intact GH-encoding chromosomal genes. This gene loss is most striking in a large chromosomal region (kw2_1427-1462) that contains 11 GHs as well as genes involved in carbohydrate transport and metabolism. Much of this region has been lost from the *cremoris* dairy starter strains. A similar chromosomal region is also found in the ssp. *lactis* strains (LLKF_1593-1630) but the lactis dairy strains do not show the same extent of gene loss (Supplementary Table 3). This clustering resembles the lifestyle adaptation region reported in *Lactobacillus plantarum* strains (Siezen and van Hylckama Vlieg, 2011), and several of the genes are likely to be involved in metabolism of plant polysaccharides. In the ssp. *cremoris* strains the cluster includes a gene encoding a secreted GH11 family endo-1,4-beta-xylanase which is the only example of a xylanase recorded in the lactic acid bacteria. Analysis of the predicted protein sequence shows that it groups with unusual xylanases isolated from uncultured organisms from arthropod guts and with xylanases from rumen bacteria (Brennan et al., 2004). Another feature of this region is the presence of arabinose metabolism genes (including a GH43 alpha-N-arabinofuranosidase) in ssp. *lactis* strains KF147, KF282, and LMG8526, and as a pseudogene in ssp. *cremoris* N41.

### Plasmid Content

Four strains with complete genome sequences (KW2, IO-1, MG1363, and IL1403) contain no plasmids, although it should be noted that MG1363 and IL1403 are plasmid-cured model organisms. To obtain an overview on the plasmid content of the other strains, we performed a dedicated sequencing effort on pooled plasmid-derived DNA. By determining the contig coverage, a prediction was made whether or not the contig was of plasmid origin. Overall, ∼11% (879 OGs) were predicted to be of plasmid origin. Among these OGs, several well-known plasmid-located features of *L. lactis* were found such as the ability to metabolize lactose, several peptidases, oligopeptide transporters and restriction-modification systems. In addition to these well-known plasmid-derived properties, we also identified 61 OGs that were not found in any of the known *L. lactis* plasmid sequences available at GenBank, several of which encode potentially industrially relevant functions (Supplementary Figure 2). For example, a four gene cluster (containing OGs 2273, 2274, 2276, and 2277) was identified to encode proteins related to capsular or exo-polysaccharide production (CPS, EPS). This gene cluster is found only in the ssp. *cremoris* strains B40, AM2, and N41 and might produce a new type of EPS. In addition, an OG annotated as nisin resistance protein (OG_3336) was found. This OG is present in SK11 SK110, AM2, and N42 and ATCC19435.

### Subspecies Disparity

**FIGURE 3:**
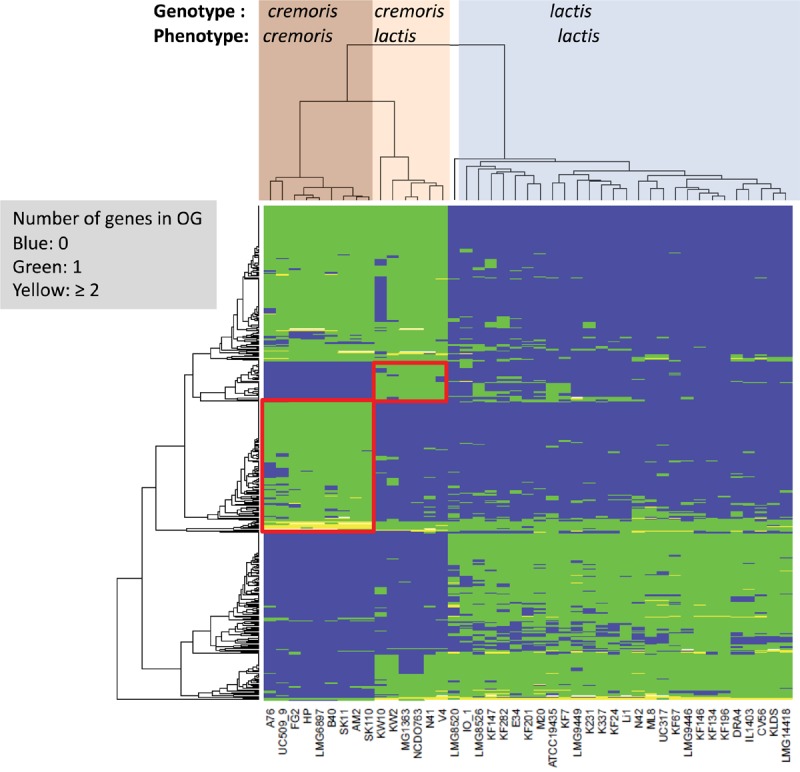
Heatmap of orthologous groups (left dendrogram) identified as most distinguishing between the different subspecies as indicated at the top of the figure (LMG8520 is the type strain *L. lactis* ssp. *hordniae*). Clusters with OGs specific to the *cremoris/cremoris* and *cremoris/lactis* groups are highlighted in red boxes. Details on the orthologous groups are given in Supplementary Table 1/Sheet 3.

The taxonomy of *L. lactis* is currently phenotypically based (Schleifer et al., 1985; van Hylckama Vlieg et al., 2006; Rademaker et al., 2007) with two main subspecies (*lactis* and *cremoris*) and one biovar (*lactis* biovar *diacetylactis*). The *lactis* and *cremoris* phenotypes are differentiated on the basis of arginine utilization, maltose utilization, growth temperature, and salt tolerance, whereas the biovar *diacetylactis* strains have the additional ability to metabolize citrate. *Lactis* phenotype strains can produce ammonia from arginine, ferment maltose, tolerate higher temperatures (40°C) and higher levels of NaCl (3%). Genotype-phenotype matching (Bayjanov et al., 2013) was used to identify the most discriminating OGs for the subspecies *lactis* and *cremoris* in this dataset (Figure 3) and allowed us to predict the subspecies with a 0% error. The analysis of the OGs that are specific for subspecies *cremoris*/*lactis* strains showed that many genes code for hypothetical proteins, regulators or transporters with unknown specificity (Figure 3).

Production of ammonia from arginine via the arginine deiminase system is the main property used to separate the two phenotypes (Reddy et al., 1969), and it was shown to play a role in acid tolerance (Cotter and Hill, 2003). This system is encoded by a cluster of nine chromosomal genes, and examination of the ssp. *cremoris* genome sequences shows that all nine genes are intact in the *lactis* phenotype strains. However, in the *cremoris* phenotype strains either arginine deiminase (*arcA*) is a pseudogene or the gene cluster contains a transposon insertion (Supplementary Table 4).

Several wild-type strains of *L. lactis* ssp. *cremoris* have been shown to produce putrescine from agmatine via the agmatine deiminase (AGDI) pathway (del Rio et al., 2015), and this may also contribute to acid tolerance. These genes are clustered and are present in 4 *cremoris*/*lactis* strains but are not found in the genomes of the *cremoris*/*cremoris* strains (Supplementary Table 4). The three gene glutamate decarboxylase (GAD) system has also been shown to be important for acid tolerance. The system is intact in most of the lactis phenotype strains, but in all the *cremoris* phenotype strains *gadB* is a pseudogene (Supplementary Table 4). The GAD system is missing from the KW10 genome, although the surrounding glutamate metabolism genes are all present.

Malolactic fermentation (MLF) is a secondary fermentation in which L-malate is converted to L-lactate and CO_2_, and is also believed to contribute to acid tolerance in *L. lactis* (Poolman et al., 1991). Three genes are involved, a LysR-family transcriptional activator (*mleR*), the malolactic enzyme (*mleS*) and a malate transporter (*mleP*). All of the *cremoris* phenotype strains contain pseudogenes in these genes (Supplementary Table 4). Based on the prevalence of pseudogenes in the these systems we predict that the acid tolerance of the *cremoris* phenotype strains is impaired and suspect that this contributes to the reduced tolerance of these strains to elevated temperatures and NaCl levels. Reduced salt tolerance has been correlated with absence or reduced activity of the betaine transporter BusA (Obis et al., 2001). The Bus operon (*busRAB*) and a similar gene cluster annotated as an osmoprotectant transport system (*cho*QS) have been lost from strains HP and FG2 but are present in the other *cremoris* strains. We could not identify a specific OG or group of OGs that might be responsible the reduced temperature and NaCl tolerance. However, there are several transcriptional regulators unique to the *cremoris* group of which some are involved in the regulation of genes involved in stress response. Regulators specific for the ssp. *cremoris* include 2 Xre-type regulators (OGs 2327 and 2368) which are known to be temperature-sensitive repressors (Wood et al., 1990) and 2 TetR-family repressors (OGs 2301 and 2412) which are described to be involved in response to osmotic stress (Ramos et al., 2005).

While the lack of maltose utilization is a phenotypic determinant of the ssp. *cremoris* we found that all *L. lactis* strains analyzed harbor full length maltose utilization genes such as maltose phosphorylase (OG_1365), trehalose 6-phosphate phosphorylase (OG_1490) and beta-phosphoglucomutase (OG_991).

The ssp. *cremoris* genomes were searched for maltose phosphorylase genes (glycoside hydrolase family 65) and this highlighted three gene clusters potentially involved in maltose transport and metabolism (Supplementary Table 5). One of these gene clusters has previously been shown to be a trehalose PTS system (Andersson et al., 2001). Most of the *cremoris* phenotype strains have pseudogenes in this gene cluster.

Maltose utilization in *L. lactis* IL1403 has been linked with a 10 gene cluster (Gabrielsen et al., 2012) that contains several glycoside hydrolases (GH13 and GH65 families) and a maltose ABC transporter (malEFG). The substrate-binding protein (malE) belongs to COG2182 which is associated with maltose transport. Most of these genes have been lost from strains HP, FG2, B40 and LMG6897, and UC509.9 contains several pseudogenes. An unusual feature is that while the glycoside hydrolase genes are orthologous, the transporter genes belong to two separate groups which show only ∼60% amino acid identity. One group includes most of the *lactis* phenotype strains along with 11 *lactis/lactis* strains, while the other group includes KW10, the *cremoris* phenotype strains and 17 *lactis/lactis* strains. Whether this difference is reflected in substrate specificity remains to be determined.

A third system potentially involved in maltose or maltodextrin utilization is found as a seven gene cluster only in the *cremoris* strains with a *lactis* phenotype and in three of 28 *lactis*/*lactis* strains (KF7, LMG8526, and ATCC19435). The genome context is the same in all these strains suggesting that these genes have been acquired as a single block. The substrate-binding protein for the ABC transporter found in this gene cluster belongs to COG1653 which is usually associated with oligosaccharide transporters.

### Lactose and Citrate Metabolism

**FIGURE 4:**
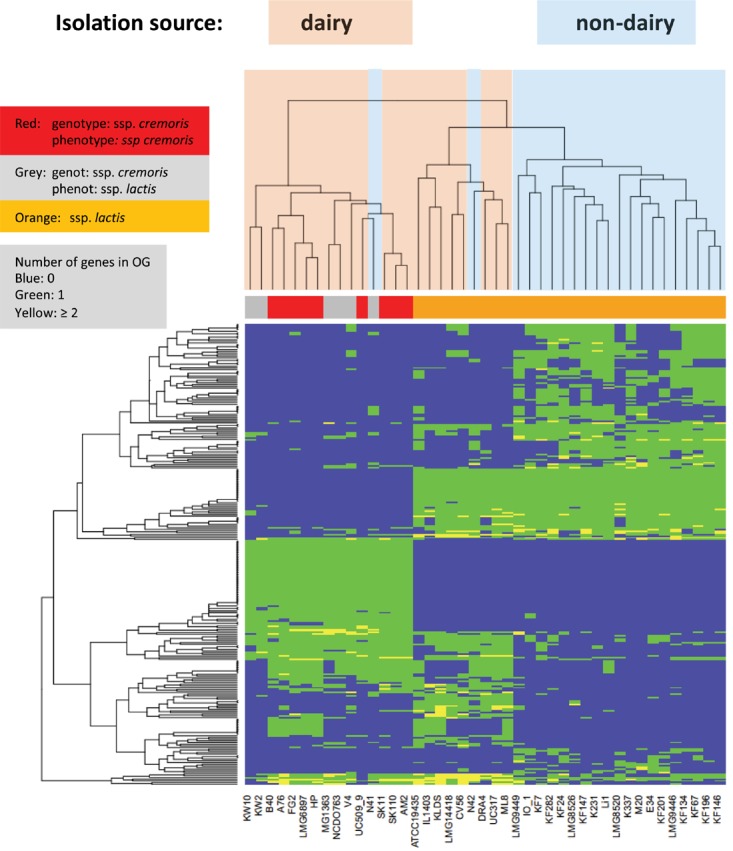
Heatmap of orthologous groups identified as most distinguishing between the strain isolation sources. Species information is given in a color bar above the heat map. Information about dairy and non-dairy origin are given by light blue and pink coloring of the tree above the heatmap. For more details see Supplementary Table 1/Sheet 4.

Lactose is taken up and phosphorylated by the lactose PTS system, and then metabolized via the tagatose pathway (de Vos et al., 1990). All genes required for uptake, conversion and regulation are organized in the *lacR-lacABCDEFGX* gene cluster, which is found to be present and complete in many *L. lactis* strains, presumably all on plasmids (high coverage contigs). All *L. lactis* ssp *cremoris* strains have this plasmid-encoded gene cluster, except for the plasmid-free strains MG1363, KW2, and KW10. Moreover, these lactose genes are also present on plasmids in *L. lactis* ssp. *lactis* strains ATCC19435, DRA4, Li-1, ML8, N42, and UC317. These ssp. *lactis* strains are isolates from dairy starters, except strains Li-1 and N42 which are grass isolates and may come from a dairy environment (Figure 4).

*Lactococcus lactis* ssp. *lactis* bv. *diacetylactis* is widely used in the food industry because it can convert citrate to aroma compounds, such as diacetyl. Citrate uptake is mediated by the pH-sensitive citrate permease P (CitP) (Sesma et al., 1990; Garcia-Quintans et al., 1998; Magni et al., 1999), which exchanges extracellular citrate for intracellular lactate. In the cytosol citrate is split into acetate and oxaloacetate by the citrate lyase complex CitCDEF (Magni et al., 1999; Martin et al., 2004). Oxaloacetate is converted to pyruvate by oxaloacetate decarboxylase (CitM). In *L. lactis* biovar *diacetylactis* strains the citrate gene cluster is on the chromosome, immediately downstream of the *als* gene encoding acetolactate synthase, which converts pyruvate to α-acetolactate, while an intact *citP* gene is found on a plasmid in a *citQRP* operon (Passerini et al., 2013; Falentin et al., 2014).

In the present study, all *L. lactis* strains were found to have the *als* gene, but only four strains (IL1403, DRA4, M20, and KF67) contain the chromosomal citrate utilization genes (OG3408-OG3414) (Supplementary Table 6). In strains IL1403 and DRA4, fragments of a citrate/malate permease (*citP/mleP*) gene were found between *citM* and *citR* while an intact *citP* gene appears to be on a plasmid in strain DRA4. *L. lactis* IL1403 is a plasmid-free *diacetylactis* strain that can no longer utilize citrate, as it has lost the pIL2 plasmid of parent strain IL594 with the *citQRP* operon (Gorecki et al., 2011). In contrast, strains M20 and K67 have an intact *citP/mleP* gene between the *citM* and *citR* genes, instead of a plasmid-encoded CitP. Since strain K67 was isolated from grapefruit juice, this appears to be an excellent example of adaptation to a niche that is rich in citrate. The citrate metabolism genes in KF67 are not found adjacent to acetolactate synthase and they are likely to have been horizontally acquired from a different lactic acid bacterium. As the different strains assigned to be of the subspecies *diacetylactis*, except for IL1403 and DRA4, are not monophyletically clustered (Figure 2) and the genes involved in citrate transport and metabolism are different in the different strains, it can be argued that the *diacetylactis* phenotype was acquired by these strains by different evolutionary events. This observation supports the current description of the strains as a dedicate biovar and not of a designated subspecies.

### Proteolytic System

The proteolytic system found in dairy starter strains of *L. lactis* consists of an extracellular, peptidoglycan-bound proteinase which cleaves milk proteins into oligo-, tri- and dipeptides. These then enter the cell via peptide transport systems and are further degraded into amino acids by dedicated peptidases (Grappin et al., 1985; Liu et al., 2010).

Most dairy starter *L. lactis* strains were found to have the *prtP* gene (encoding extracellular serine proteinase PrtP) and adjacent *prtM* gene (encoding its maturase PrtM) located on a plasmid (Supplementary Figure 3). An intact *prtP* gene was found only in strains NCDO763, SK11 (Siezen et al., 2005), SK110, AM2, UC509.9 (Ainsworth et al., 2013), N41, N42, and UC317. In strains FG2, LMG6897, B40, and ML8 the *prtP* gene is present but was not recognized by the Prokka annotation system, because the *prtP* gene was fragmented on different small contigs. The *prtP* gene is known to have repeats near its C-terminus, which could cause sequence assembly problems. In addition, two different chromosome-encoded intracellular serine endoproteinases were found (OG_4412, OG_4413); in strains ATCC19435, N42, and LMG9447, and a different one in strain CV56.

Nearly all intracellular peptidases of known specificity (Liu et al., 2010) are chromosome-encoded; these are present and complete in all *L. lactis* genomes: PepC, PepN, PepM, PepA (pseudogene in KF147), PepV, PepT, PepXP, PepQ, and PepP. Two paralogs of PepD1, encoding a dipeptidase, are present in all strains, but one variant is a pseudogene in strains AM2, SK110, SK11, and LMG8520. A plasmid-located *pcp* gene, encoding pyrrolidone-carboxylate peptidase, is only present in most ssp. *cremoris* strains (except LMG6897, A76, KW2, KW10, and V4), but located on the chromosome of strain MG1363. Absent in all genomes are the peptidases PepE/PepG, PepI, PepR, and PepL, which are more commonly found in other lactic acid bacteria (Liu et al., 2010). Another five peptidases of unknown specificity are present in all strains.

Two paralogs of PepF, encoding oligoendopeptidase F, are found based on differences in amino acid sequence. All strains except ATCC19435 contain a chromosomally encoded PepF (pseudogene in AM2, SK110, and KLDS). An additional plasmid-encoded PepF is found in most ssp. *cremoris* strains (absent in LMG16897, V4, KW2, KW10; pseudogene in AM2, SK110) and in ssp. *lactis* ATCC19435. Two paralogs of PepO, encoding neutral oligoendopeptidase O, are found in most strains. They are difficult to distinguish since their amino acid sequences are nearly identical, and that could lead to assembly difficulties. A chromosome-encoded PepO paralog appears to be present in all ssp. *cremoris* and many ssp. *lactis* strains, but appears to be a pseudogene in several strains. The presence of an intact *pepO* gene is generally linked to the presence of a complete *oppACBFD* operon encoding the ABC transporter for uptake of oligopeptides. A plasmid-located *pepO* paralog is present in most ssp. *cremoris* strains (absent in KW10, KW2), and is directly adjacent to an additional *opp* operon. *L. lactis* ssp. *lactis* strains appear to have only one *opp* operon, either on the chromosome or on a plasmid, and it many cases it is not clear whether this transporter is functional due to putative pseudogenes. All *L. lactis* strains have a chromosome-encoded di/tripeptide transporter DtpT (pseudogene in ATCC19435, LMG8520). The advantage of having multiple genes for certain proteolytic functions is assumed to provide more efficient utilization of peptides derived from milk proteins.

### Bacteriocins – Nisin

A complete nisin biosynthesis cassette *nisABTCIPRKFEG* (Kuipers et al., 1993) is present on the chromosome of ssp. *lactis* strains CV56 and IO-1, and is flanked by transposase fragments. A complete *nis* gene cluster was found, at the same chromosomal insert position as in strain CV56, in 11 ssp. *lactis* strains: KF134, KF146, KF196, KF282, K231, KF24, K337, KF67, KF7, Li-1, and LMG8526. All the strains of plant/vegetable origin can produce nisin Z. Four strains (V4, LMG14418, LMG9446, and KF147) have an incomplete chromosomal *nis* gene cluster and cannot produce nisin, but they have retained some immunity genes (i.e., *nisFEG* and/or *nisI*). *L. lactis* ssp. *cremoris* strains FG2 and N41 have a fragment of the *nis* gene cluster encoding only *nisP, nisI* and a truncated *nisC*, which should also confer nisin immunity; this organization is typical of plasmid localization (Tarazanova et al., 2016).

### Novel 2-Component Lantibiotic

One of the contigs of the genome of *L. lactis* ssp. *lactis* KF146 is a 37-kb plasmid fragment (high sequence coverage) that contains a 7-gene cluster encoding a class I 2-component lantibiotic. This gene cassette is very similar to the *smbM1FTM2GAB* cluster in *Streptococcus mutans*, in which SmbA and SmbB are lantibiotic precursors with similarity to lacticin A1 and A2 (Yonezawa and Kuramitsu, 2005). We have adopted the same nomenclature, whereby the *L. lactis* bacteriocin genes *llbM1* and *llbM2* encode lantibiotic biosynthesis proteins involved in dehydration and cyclization of the lantibiotic precursors, while *llbF, llbT*, and *llbG* encode components of an ABC transporter, of which LlbG has an N-terminal peptidase domain for cleavage of the pre-peptide and activation of the lantibiotic subunits. Similar gene clusters are present in various strains of *S. mutans, S. gallolyticus, S. suis*, and *S. rattus* (Hyink et al., 2005; Hinse et al., 2011). The lantibiotic precursor LlbA is 52% identical to SgbA, and 46% to SmbA found in several *S. mutans* strains, while LlbB is 65% identical to SgbB, 37% to SmbB, and 68% to the SsbB of *S. suis* (Supplementary Figure 4).

### Non-ribosomal Peptide (NRPS)/Polyketide (PKS) Synthesis

A NRPS/PKS gene cluster has been identified in *L. lactis* KF147 (Siezen et al., 2008, 2010a). It was hypothesized that the NRPS/PKS product in *L. lactis* functions in microbe–plant interactions (defense or adhesion) or that it facilitates iron uptake from the environment. In the present study, this complete NRPS/PKS gene cluster is found to be present in five other *L. lactis* strains, i.e., the plant strains KF147, KF146, KF134, KF196, and Li-1 (Supplementary Table 7). In each strain the gene cluster has been inserted at the same position on the chromosome downstream of the acetolactate synthase gene. It is likely that this region is a hotspot for gene insertion as the citrate metabolism genes are found at the same location in biovar *diacetylactis* strains. There is little sequence diversity between these NRPS/PKS gene clusters in these *L. lactis* strains, suggesting that they are recently acquired from the same unknown host.

A highly similar NRPS/PKS gene cluster occurs in many *S. mutans* strains (Aikawa et al., 2012). The order of the genes is exactly the same as in *L. lactis*, and the individual proteins are 69–74% identical to those of *L. lactis*. Therefore, although the function of the NRPS/PKS product in these two species is likely to be the same, the gene cassette was probably not recently horizontally transferred between species. In *S. mutans* UA140, the NRPS/PKS locus was demonstrated to produce a metabolite that contributes to oxidative stress tolerance and biofilm formation (Wu et al., 2010). As *L. lactis* KF147 was recently shown to express this locus during growth in association with plants, this could indicate that the end metabolite might confer protection against reactive oxygen species encountered in plant fermentations as well as on living plant surfaces (Golomb and Marco, 2015).

### Exopolysaccharides (EPS)

Lactococcal cell-wall polysaccharides decorate the peptidoglycan network, and in some cases form a thin outer layer termed the polysaccharide “pellicle” (PSP) (Delcour et al., 1999; Kleerebezem et al., 2010). These polysaccharides have been implicated in bacteriophage recognition and attachment (Forde and Fitzgerald, 1999; Dupont et al., 2004; Mahony et al., 2013; McCabe et al., 2015). *L. lactis* has two known chromosomal loci for cell-wall polysaccharides biosynthesis (called RGP and EPS cluster) and one gene cluster for teichoic acid biosynthesis (Siezen et al., 2011). These regions show a lot of variation in gene order and composition between *L. lactis* strains. In a few strains an EPS biosynthesis cluster can be found on plasmids. This variability of cell-wall polysaccharide genes suggests a rich variety in structures of the produced exopolysaccharides in these *L. lactis* strains.

Rhamnose is one of the major components of these exopolysaccharides (Sijtsma et al., 1991; Looijesteijn et al., 1999). L-dTDP-rhamnose is formed in a four-step enzymatic reaction from glucose 1-phosphate, which involves the activities of glucose-1-phosphate thymidylyl transferase, dTDP-glucose-4,6-dehydratase, dTDP-4-keto-L-rhamnose-3,5-epimerase, and dTDP-L-rhamnose synthase, encoded by the genes that are commonly designated *rfbABCD*, respectively (Shibata et al., 2002).

The RGP gene cluster for biosynthesis of rhamnose-glucose polysaccharides, also called CWPS (cell wall-associated polysaccharide) gene cluster, appears to consist of three subclusters (Dupont et al., 2004; Mahony et al., 2013; Sadovskaya et al., 2017). The first cluster contains the *rmlA-D* (or *rfbA-D*) rhamnose biosynthesis genes, which are found to be highly conserved in all *L. lactis* strains. The second cluster contains the *rgpABCDEF* genes, which encode polysaccharide biosynthesis and export, including the priming glycosyltransferase RgpA and ABC transporter subunits RgpC and RgpD. The exact function of *rgpE* is still unclear (Shibata et al., 2002). The *rpgA-D* genes are also highly conserved in all *L. lactis* strains. Large differences between strains are found in the third sub-cluster which encodes a variety of different sugar transferases, thereby highlighting the diversity and complexity of lactococcal polysaccharides. Based on the presence and absence of all genes found in these RGP/CWPS clusters, three genotypes (A, B, and C) were first defined (Mahony et al., 2013), while C-genotype strains were further grouped into 5 subtypes (Ainsworth et al., 2014a). Polysaccharide structures of members of CWPS classes A, B, and C have recently been determined (Ainsworth et al., 2014a; Vinogradov et al., 2018a,b). In our present study, the composition of the RGP gene clusters in the 43 *L. lactis* strains allows a tentative assignment of the produced polysaccharides to the CWPS subgroups A, B, and C (Supplementary Table 8/Sheet 2 and Table 9).

The second biosynthesis cluster is the so-called “EPS cluster” of 13 genes *epsXABCDEFGHIJKL*, as in the plant-derived strain KF147 (Siezen et al., 2008, 2010a, 2011). In this cluster the *epsA* and *epsB* gene products determine the length of the EPS and are essential for the biosynthesis of EPS. The *epsC* gene product is not necessary for the biosynthesis and plays a role in regulation of phosphorylation of the *epsB* protein. EpsD is the priming glycotransferase, which is anchored in the membrane and essential for the production of EPS (Boels et al., 2004). EpsI is believed to be responsible for the polymerization, and *epsJ* and *epsK* gene products play a role in the export of EPS.

Most *L. lactis* strains do not contain an EPS gene cluster. The EPS cluster similar to strain KF147 was only found (in a conserved position on the chromosome) in seven other *L. lactis* strains, all plant isolates. Clusters with similar EPS genes are found in 10 other strains, mainly ssp. *cremoris*, but are located elsewhere on the chromosomes or on plasmids (Supplementary Table 8/Sheet 1). Putative plasmid-located EPS clusters are present in strains B40 [on pNZ4000 (van Kranenburg et al., 1997, 2000), AM2, and N41]. Of the chromosomal EPS cluster the *epsX, epsA-D, epsK*, and *epsL* gene are highly conserved in these strains. Only in strain LMG9446 the *epsA-D* and the *epsX* genes are absent. The genes *epsE-J* are highly variable between strains. Only strains KF147, KF146, and KF196 have an identical EPS cluster.

In *L. lactis* one teichoic acid (TA) biosynthesis gene cluster is known (Supplementary Table 8/Sheet 3). This cluster is completely absent in 7 *L. lactis* strains. In other strains the number of genes varies between 4 and 18; several groups can be made, based on the presence and absence of these genes. Of these groups, the *cremoris/cremoris* genotype/phenotype strains presumably do not have a functional TA biosynthesis, since most genes are absent or pseudogenes, and the essential export genes *tagG* and *tagH* are absent. These appear to have been replaced by transposases.

### Prophages

Bacteriophages are the leading cause of fermentation problems in the dairy industry, with *L. lactis* phages and the identification of phage-resistant strains being a focus of study since the 1930s (Lawrence et al., 1978). Many *L. lactis* strains are known to be lysogenic, and analysis of eight complete genome sequences for P335-type prophages identified seven chromosomal integration locations (Kelly et al., 2013). The presence of prophage or prophage remnants at these locations in the 43 genomes is shown in Supplementary Table 10. The 43 genomes were also searched for prophage-specific genes (encoding portal or terminase proteins) which resulted in identification of a further five integration locations (Supplementary Table 10). Of the 43 genomes, 42 contain between one and five prophages. The most common location for phage integration is site 6 (23/43 strains) between the *sun*L and *fmt* genes, with all subspecies *cremoris* dairy starter strains having a prophage or a prophage remnant at this location. No prophage sequence could be identified in KW10 which originated from fermented corn. Kelleher et al. (2018) recently examined 30 complete *L. lactis* genomes and found a similar distribution of prophage sequences, although they did not check their genomic context. It can be concluded that prophages are an integral part of the genome of most *L. lactis* strains, and that they have co-evolved with their bacterial host. The inability of most *L. lactis* prophages to be induced (Kelleher et al., 2018) suggests they are stable residents within the genome, although they retain the potential to exchange genes with other P335-type phages or to mediate rearrangements of the bacterial chromosome (Kelly et al., 2013).

### Protein Secretion Systems

Protein secretion has a key role in determining how bacteria interact with their environment and in lactic acid bacteria the majority of proteins are secreted by the conserved Sec pathway. However, several other bacterial secretion systems are known, and the genome of strain KW2 (isolated from fermented corn) encodes two additional systems which have not previously been described in this species.

SecA2 system: KW2 has a gene cluster (kw2_0790-_0804) located between *mut*Y (OG_1194) and *pep*V (OG_0624) that make up an accessory SecA system similar to those found in streptococci and several other Gram-positive species (Bensing et al., 2014). In KW2 these genes are predicted to encode proteins involved in the glycosylation and export of a large (2338aa) serine-rich glycoprotein (Supplementary Figure 5). The secreted protein has an atypical signal sequence (TIGRFAM number TIGR03715) and a LPXTG-motif C-terminal cell-wall anchor. KW2 is the only strain where this gene cluster appears intact but remnants of this locus are present at the same location in all the *L. lactis* ssp. *cremoris* strains as well as in the ssp. *lactis* strains M20, KF201, K337 and LMG8520.

Ess (ESX secretion) pathway: ESX protein (type VII) secretion systems were initially identified in *Mycobacterium tuberculosis*, and have subsequently been found in a variety of Gram-positive bacteria (Unnikrishnan et al., 2017). KW2 has a gene cluster (kw2_1252-_1268) that includes the secretion proteins EssA-C and a WXG100 family protein that characterize this category of secretion system (Supplementary Figure 5B). In KW2 this gene cluster is found next to the prophage integrated between *suf*B and *fab*L. Remnants of this locus are present at the same location in all the *L. lactis* ssp. *cremoris* strains, and found next to a prophage in strains A76 and V4. The ssp. *lactis* strain M20 (isolated from soil) has a similar but shorter (10 genes compared to 17 in KW2) intact ESX system at the same location.

The role of these secretion systems in *L. lactis* and the proteins they secrete is not known, but in other organisms similar systems are known to be involved in bacterial colonization and adhesion, and in several cases play an important role in bacterial pathogenesis (Bensing et al., 2014; Unnikrishnan et al., 2017). Curiously, there have been a small number of reports of human infections caused by *L. lactis* ssp. *cremoris* (Hadjisymeou et al., 2013), some of which also make a link to the consumption of unpasteurized dairy products. None of the infection-associated strains have had their genomes sequenced, but it would be valuable to know if these secretion systems occur in these strains.

### Sex Factor

The sex factor of *L. lactis* is a chromosome-located mobile genetic element involved in high-frequency conjugation, which has previously been identified in *L. lactis* strain NCDO712 and MG1363 (Gasson et al., 1992; Godon et al., 1994; Wegmann et al., 2007). It can excise from the chromosome and was found to form plasmid co-integrates, e.g., with the lactose plasmid pLP712 (Gasson and Davies, 1980; Walsh and McKay, 1982; Godon et al., 1995). Sex factor integration and excision is site-specific involving an identical 24 bp sequence on both the sex factor and the chromosome. The sex factor encodes a relaxase MobD which can nick duplex DNA and is essential for horizontal transfer of the element. Based on the presence of a *mobD* gene, excisionase/integrase and a 24-bp repeat sequence at the boundaries, we identified regions resembling a chromosome-located sex factor in *L. lactis* strains MG1363, NCDO763, ATCC19435, N42, KLDS, E34, and IO-1 (Supplementary Table 11). These regions varied considerably in size (about 40–80 kb), chromosomal insertion position, and composition of encoded functions. The sex factor of strain NCDO763 is basically identical to that of strain MG1363, but lacks 12 consecutive genes including a 6-gene *ter* operon encoding a membrane-associated stress response complex (Anantharaman et al., 2012). The sex factor of MG1363/NCDO763 encodes a cell-membrane-anchored protein CluA that facilitates cell-to-cell contact and can cause a cell-aggregation/clumping phenotype (Godon et al., 1995), but this gene is absent in the other sex factors.

The 71-kb sex factors of strains ATCC19435 and N42 are entirely identical, but share only 10 orthologs with strain MG1363. The 83-kb element of strain KLDS resembles the sex factor of the latter two strains (35 shared orthologs). These sex factors of strains ATCC19435, N42, and KLDS each encode abortive infection proteins AbiGI and AbiGII, and are flanked by genes encoding an excisionase and an integrase, which is typical of a transposon-like element. A putative sex factor on a 66-kb contig of strain LMG8526 shares only 20 orthologs with strain MG1363; this contig has a higher sequence coverage and encodes various plasmid replication proteins, suggesting it could be a plasmid co-integrate instead. Putative sex factors in the plasmid-free strains E34 and IO-1 are smaller but similar and share 25 orthologs.

All these putative chromosomal sex factors carry the essential relaxase *mobD* gene, which is interrupted by a group II intron (Dunny and McKay, 1999) in strains MG1363, NCDO763, and KLDS, but not in the other strains. The *mobD* gene with group II intron also appears to be present on plasmids in all dairy *L. cremoris* strains and in *L. lactis* UC317. The group II intron encodes a reverse transcriptase/maturase LtrA which plays an enzymatic role in splicing and genetic mobility.

### Phenotype/Genotype Matching

A large number of the strains described in this study have been extensively studied for the presence of different phenotypes related to carbon source utilization, metal resistance and antibiotic resistance (Bayjanov et al., 2013). These phenotypes were previously matched with genotype information based on comparative genome hybridization (CGH) data obtained from microarrays containing the genes of a selected number of reference strains [IL1403, MG1363 and incomplete genomes of KF147 and KF282 (Bayjanov et al., 2009)]. In the original study, several phenotypes could be matched with genetic differences in the strains, explaining the observed phenotype. For example, genes were identified that are associated with the ability of the strains to grow on arabinose, sucrose, lactose and melibiose as well as the resistance to copper and arsenite (Bayjanov et al., 2013).

We here re-examined the phenotypes from Bayjanov et al. (2013) by matching them with the draft genome sequences. As the genome data is more comprehensive compared to the CGH data from the previous study, this should allow to find more genotype/phenotype matchings.

#### Carbon Source Utilization

In addition to the phenotypes described in the Bayjanov paper, we identified 5 more phenotype/genotype matches with other carbon sources. For gentiobiose, starch, ribose and salicin, clear matches were found with genes in the genomes that have a link with carbon source utilization. For gentiobiose, which is a glucose disaccharide, several carbohydrate utilization genes were found to be correlated with the phenotype; an uronate isomerase (EC 5.3.1.12), xylulokinase, aldose-1-epimerase and a xylose transporter. Interestingly, apart from the xylose utilization genes, all the genes are located on different locations on the genome. Within the xylose gene cluster, multiple other genes related to xylose degradation were found, including a beta-xylosidase. If this gene cluster could also (or primarily) act on gentiobiose would need to be validated.

For both starch and ribose, we identified a ribokinase among the best scoring hits in the gene-trait matching. Interestingly, this ribokinase is actually found in most strains, but truncated in four strains (AM2, FG2, HP, and LMG6897) and absent in LMG8520. The only strain with a complete copy of the ribokinase that was not positive for growth on ribose was strain SK11, which could point at another gene being absent or disrupted in this strain. In-depth analysis on the ribose metabolism genes showed that the permease component of the ribose ABC transporter (OG_1649) has been truncated in SK11. The genotype/phenotype relation found in this particular case was not identified in the original study because of the truncation of the gene, causing it to still be identified in the CGH as present. Strain KF7 is also unable to grow on starch, although the ribokinase and the ABC transporter are present on the genome.

Growth on salicin is correlated with 15 genes with approximately the same presence/absence pattern. Among these genes is a cluster of sugar ABC transport genes present in combination with an alpha-mannosidase. This transporter could well function as a transport system for salicin, while the mannosidase could cleave the salicyl alcohol from the glucose. Alternatively, salicin could be degraded by GHI family glycoside hydrolases. Several of these systems have been identified in the on different locations on the genomes of the different *L. lactis* strains.

#### Heavy Metal Resistance

The original phenotype/genotype matching paper already describes the presence of copper and arsenite resistance genes in the genomes of several *L. lactis* strains. Those observations were confirmed when performing the GTM on the genome sequences. In addition, we could identify a set of genes related to the resistance of cadmium, another heavy metal that was described in the results of Bayjanov et al. (2013). The highest scoring gene in that gene-trait matching is annotated as a cadmium-transporting ATPase. Combined with an efflux accessory protein, which is also among the top-10 scoring genes, this transport system could function as a highly efficient cadmium resistance system. Manual inspection of the GTM results also pointed out why this genotype to phenotype matching was not successful in the original publication, as these transport genes are not found in the reference genomes present on the CGH microarray.

## Discussion

The comparative analysis of 43 *Lactococcus lactis* genomes revealed ∼ 7800 (orthologous groups and highlights the extensive pangenome of this species). This is considerably higher than the 3877 OGs found in an earlier comparative genome hybridization study that used many of the same strains (Bayjanov et al., 2010, 2013; Siezen and van Hylckama Vlieg, 2011), but used arrays based on the sequences of only 5 strains. The pangenome size reported here is also almost 1900 OGs larger than that reported by Kelleher et al. (2017) which was based on the genome sequences of 30 lactococcal strains. In their analysis, plasmids were not included in the study. Out of the 7800 orthologous identified in our study, 879 were of plasmid origin, which explains part of the discrepancy between our analysis and the Kelleher manuscript. In their study 22 out of the 30 strains were dairy isolates. Our study includes 24 non-dairy isolates and the larger pangenome size found here is presumably caused by additional OGs found in the non-dairy strains. As the pangenome of *L. lactis* is much larger than that of other lactic acid bacteria (Smokvina et al., 2013) the question arises if this is the result of horizontal gene transfer or a relatively broad species definition. The analysis of the pangenome per subspecies showed that their sizes are 4968 and 6545 OGs for ssp. *cremoris* and ssp. *Lactis*, respectively, arguing for *L. lactis* having a rather broad species definition. Another difference in the assessment of the pan- and core-genome as presented here compared to the Kelleher study is that our pangenome levels off toward a maximum of OGs while it seems to keep on increasing in the Kelleher paper. One of the explanations between the difference in pangenome graphs could be that the genomes used here represent a broader population of *L. lactis*, resulting in more overlap in gene content between individual genomes. Another difference between the analyses in the two studies was that all genomes compared in this study were re-annotated with the same pipeline. This results in less bias in the gene calling between the different genomes and more overlap in shared genes.

The broad occurrence of plasmid DNA especially in the dairy strains of *L. lactis* resulted in ∼10% of OGs being associated with plasmids. The observation that plasmids encode several functions that enable growth in milk makes them highly relevant for fermentation applications. While on one hand it makes sense that dairy relevant traits like lactose utilization are on mobile elements it is surprising that they have not been integrated into the genome in any of the analyzed strains. It would be interesting to trace the origin of the genes that mediate lactose fermentation with *Enterococcus* being the most likely candidate.

The documented discrepancy between the genotype and the phenotype of the subspecies *cremoris* and *lactis* is still unresolved at the genome level and in a recent paper by Kelleher et al. (2017) the authors suggest that the solution to this might not be possible without the use of transcriptome and/or metabolome data. Our analysis shows that we were able to fully distinguish the true *cremoris* strains being a subclade of the complete *cremoris* subspecies in the set of strains used. Genes that are specifically lost (or are considered as pseudogene) in the true *cremoris* subclade include a maltose transport system and transcriptional regulators that are described to be involved in osmotic and temperature stress, which are properties used to distinguish the *lactis* and *cremoris* subspecies at a phenotypic level.

The development of dairying that followed the domestication of ruminant animals has had a major impact on the selection and evolution of sheep, goats and cattle (Larson and Fuller, 2014) and forage plants (Glémin and Bataillon, 2009). Humans have also been affected as evidenced by studies of the spread of genes for lactase persistence (Leonardi et al., 2012). It is believed that Neolithic humans would have been unable to digest the lactose in milk and so fermented dairy products would be an early development (Curry, 2013) with the earliest evidence for cheese making dating from the 6th millennium BC (Salque et al., 2013). *Lactococcus lactis* is a key organism involved in dairy fermentation and the strains used as industrial cheese starter cultures also show evidence of domestication. This domestication is best shown by *L. lactis* strains with the *cremoris* phenotype. These have no documented counterpart in nature but by comparing the genomes of dairy and non-dairy strains we provide evidence of the genetic events that have shaped their evolution resulting highly specialized dairy strains. Generally, the wild relatives of industrially used microbes have not been studied in detail, but there is interest in how these valuable organisms arose and how they may be further improved. The dairy strains have gained the ability to use casein and lactose through acquisition of plasmid-encoded genes, but at the cost of an extensive multiplication of mobile genetic elements resulting in pseudogenes and the loss of numerous functions. Similar expansion of IS elements has been observed in host-restricted pathogens (Mira et al., 2006) where it is accompanied by loss of genes by deletion. The evolutionary outcome of this is that now that many genes are not needed they become dispensable and are lost or degraded, a process described as reductive evolution (Kelleher et al., 2017). In this comparison we have focused on accessory protein secretion systems, carbohydrate utilization, exopolysaccharides and other genes that give rise to the *cremoris* phenotype. In all categories the genome of the dairy starter strains appears extensively degraded predominantly as a consequence of insertion and deletion of mobile genetic elements. Not only have insertions and deletions occurred but there are also changes in gene order through inversions or translocations of parts of the lactococcal chromosome (Kelly et al., 2010). Clustering of these strains suggest that the industrial used starter cultures have all evolved from a small number of lineages as has often been suggested from phage work. The population of *cremoris* phenotype is small and these strains can be seen as true domesticated microbes whose genome is now so degraded that they seem restricted to a man-made environment. The development of starter cultures and their industrial selection and use over the last century has contributed to this specialization. These observations explain why it has not proved possible to isolate novel *L. lactis* ssp. *cremoris* strains with properties similar to dairy starters from environmental sources.

Overall the analysis of full genome sequences of a diverse set of *Lactococcus lactis* strains allowed us to identify niche and subspecies specific genes that could not be identified earlier. Besides the uncovering of evolutionary relationships, the analysis of functional properties is anticipated to be useful for industrial strain discovery and selection processes.

## Data Availability Statement

The datasets generated for this study can be found in Zenodo, https://doi.org/10.5281/zenodo.1471674.

## Author Contributions

MW, RS, and HB conceived the study. All authors analyzed the data. MW, RS, WK, and HB wrote the manuscript.

## Conflict of Interest Statement

MW, SvH, and HB are employed by NIZO Food Research B.V. The remaining authors declare that the research was conducted in the absence of any commercial or financial relationships that could be construed as a potential conflict of interest.

## Footnotes

1 https://img.jgi.doe.gov/

2 https://cran.r-project.org/bin/windows/base/
