# Supplementary material for: Comparative Genome Analysis of Lactococcus lactis Indicates Niche Adaptation and Resolves Genotype/Phenotype Disparity
Source: Front Microbiol. 2019 Jan 31;10:4. doi: 10.3389/fmicb.2019.00004 (PMC6365430; doi:10.3389/fmicb.2019.00004)
Supplement: Supplementary file 1 [file Data_Sheet_1.ZIP › Wels et al._Supplementary Material.pdf]

Supplementary Material

Comparative genome analysis of *Lactococcus lactis* indicates niche adaptation and resolves genotype/phenotype disparity

Michiel Wels[1,2], Roland Siezen[2,3,4], Sacha van Hijum[1,2,3], William J. Kelly[5], Herwig Bachmann[1,2,6*]

[1] NIZO B.V., Ede, The Netherlands

[2] TI Food and Nutrition, Wageningen, The Netherlands

[3] Centre for Molecular and Biomolecular Informatics, Radboud Institute for Molecular Life Sciences,

Radboud UMC, Nijmegen, The Netherlands.

[4] Microbial Bioinformatics, Ede, The Netherlands.

[5] Ashhurst, New Zealand

[6] Systems Bioinformatics, VU University, Amsterdam, The Netherlands

[*]Correspondence: Herwig Bachmann, NIZO B.V., P.O. Box 20, 6710BA Ede, The Netherlands; M: +31 318 659652; E: Herwig.Bachmann@nizo.com

Supplemental Figures

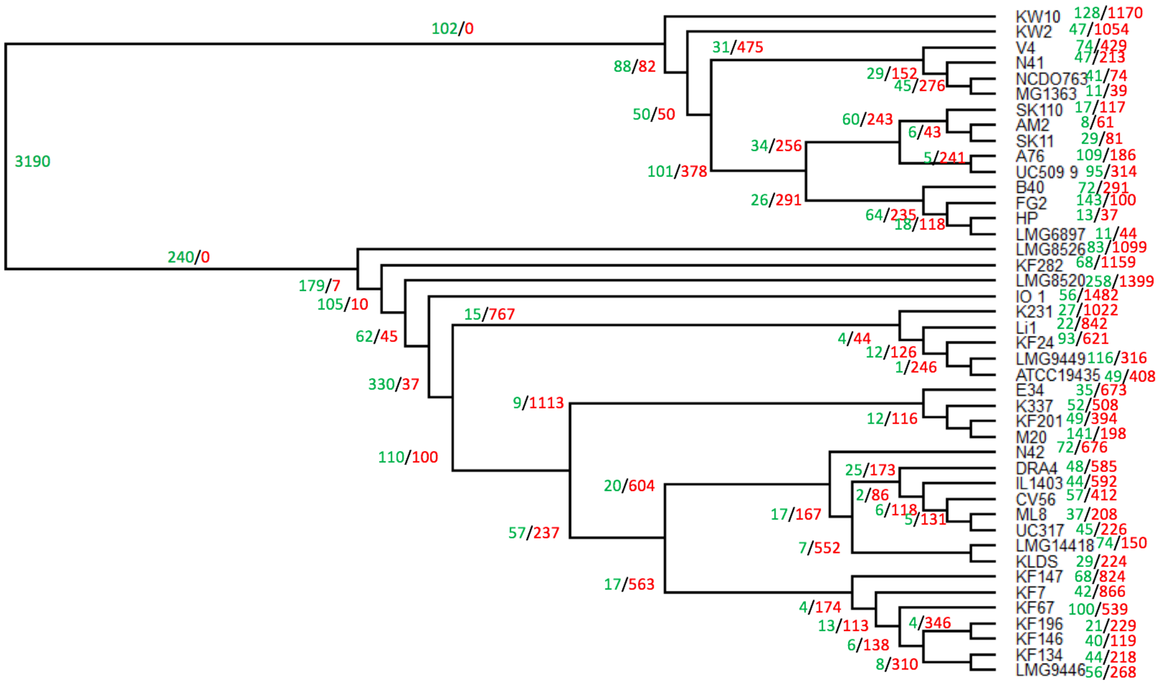

Supplementary Figure 1: Phylogenetic tree based on the single nucleotide polymorphisms (SNPs) in the *L. lactis* core genes. Gene gain and gene loss at the individual nodes are given in green and red respectively.

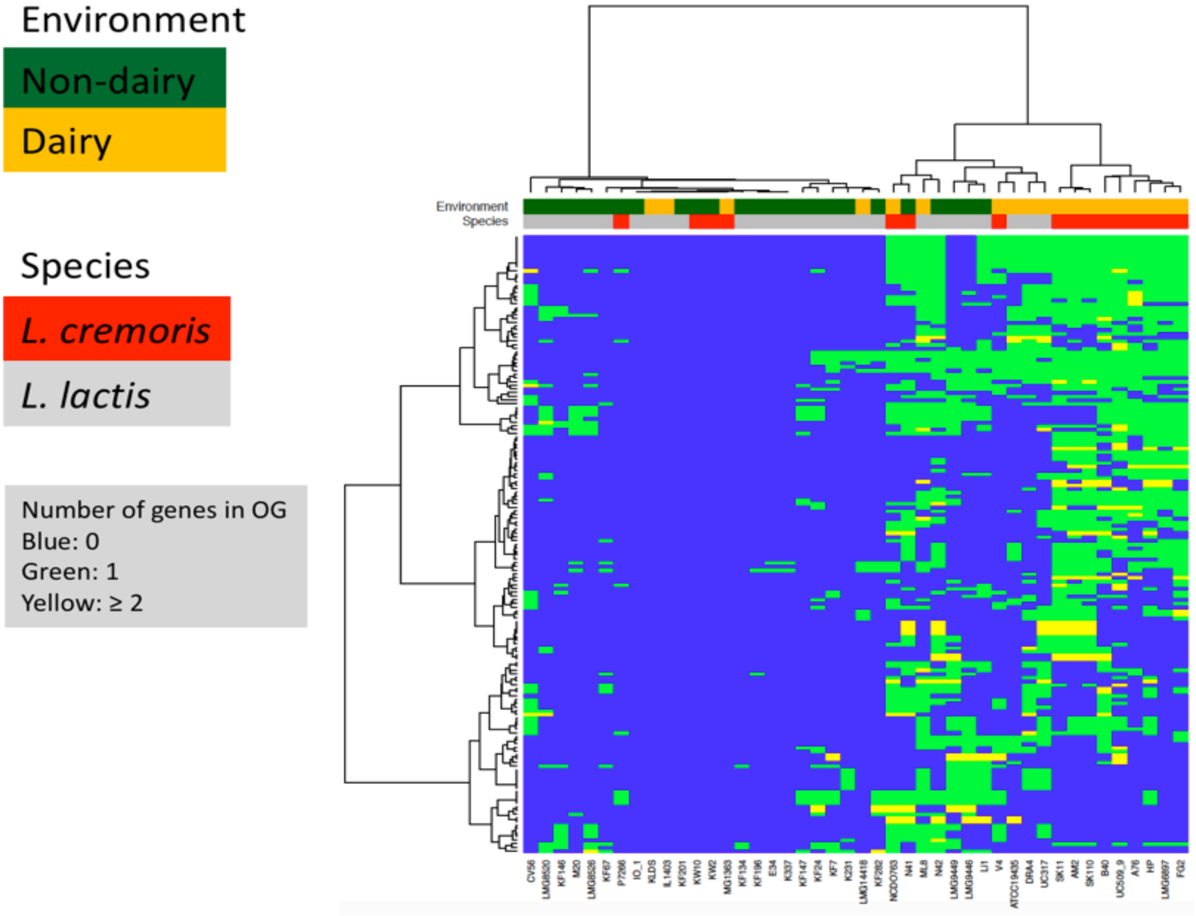

Supplementary Figure 2: Heatmap of orthologous groups (left dendrogram) identified as plasmid encoded genes. Isolation environment and species information are indicated at the top of the figure (LMG8520 is the type strain *L. lactis* ssp. *hordniae*). Each row represents one OG. Each column represents one strain. For details see Supplementary Table 1/Sheet 5.

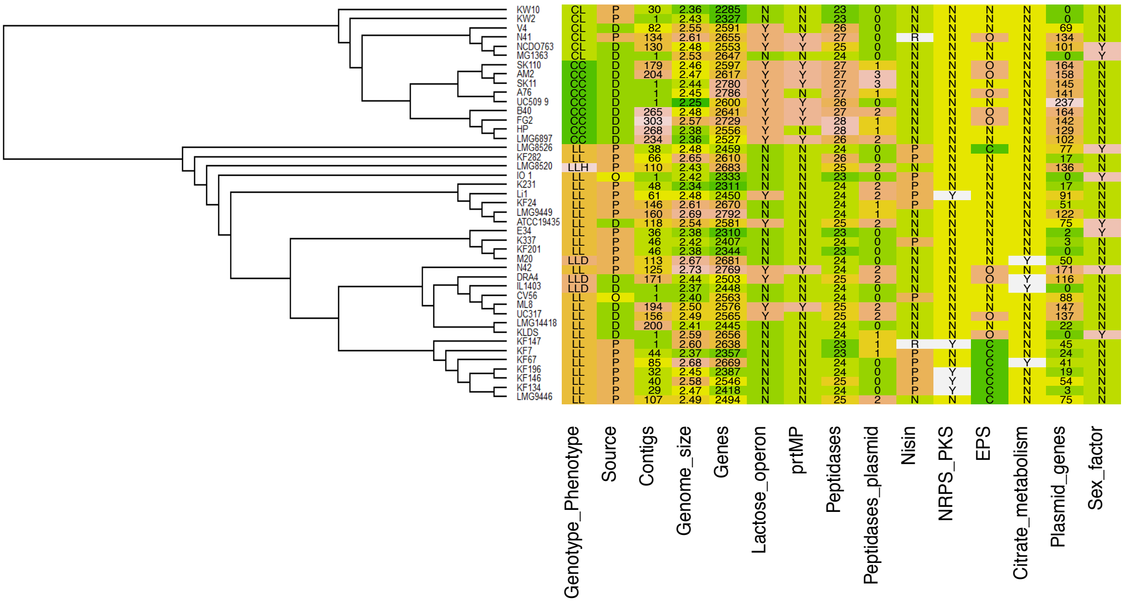

Supplementary Figure 3: Phylogenetic tree and its relation to a selected set of phenotypic properties. Abbreviations: Genotype-Phenotype CL=*cremoris-lactis*, CC=*cremoris-cremoris*, LL= *lactis-lactis*, LLH=*lactis-lactis-hordniae*, LLD=*lactis-lactis-diacetylactis*; Source D=dairy, P=plant material, O=other; Nisin P=producer, R=resistant, N-contains no genes from the nisin cluster; EPS C=conserved on chromosome, O=other position on chromosome or plasmid-located, N= contains no EPS genes; in all other columns y=yes/present, n=none/absent. The branch of the dendrogram on the length does not reflect the phylogenetic distance (see Figure 2 for phylogenetic distance). Colors are only for easier visualization of differences within columns.

```
-SgbB:  MNEFKKIGLENELFNLI-EDTEQLE-LMSGAGWTGVAKSITSCSLLSIGLGNDGWVCTWTAECQATCR

-LlbB:  MKNLKNSGLQEDYLSLI-EETKDLE-LISGAGWGGVAKSITSCSILSITLGNNGWICTWTAECQKGCK

-SmbB:  MKEIQKAGLQEE-LSILMDDANNLEQLTAGIG-TTVVNS-----TFSIVLGNKGYICTVTVECMRNCSK


-SgbA: MEHNNLLNISEEEMQKNMVLLIEDEDQELSGASTPGCAWAAVSAISTVSALFQITTACTTRCYHP

-LlbA: MKNNNLLDVTDEVMEKNIVTLIEDEDLNISGAGTPA-GITVV---ITIGGLLQVTTACTSRCWHP

-SmbA: MKSNLLKINNVTEMEKNMVTLIKDEDMELAGGSTPACAIGVV---GITVAVTGISTACTSRCINK
```

Supplementary Figure 4: Sequence alignment of the lantibiotic precursors A and B of *L. lactis* (Ll), *S. mutans* (Sm), and *S. gallolyticus*. Identical residues are colored green, while Cys residues probably involved in cyclization are colored yellow.

A. SecA2 system

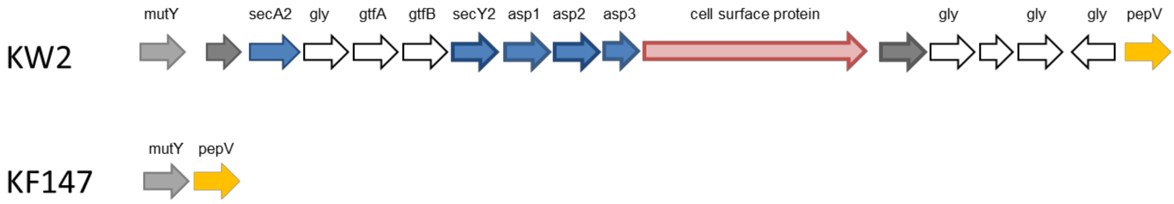

B. ESX system

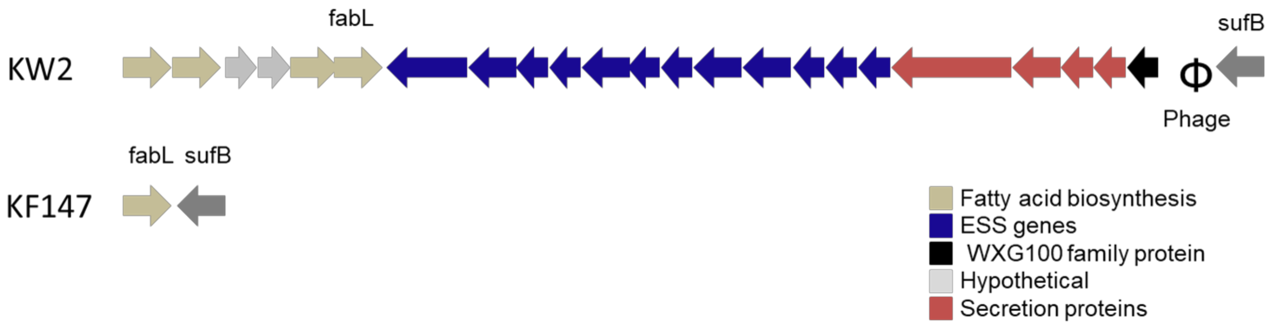

Supplementary Figure 5: Accessory protein secretion systems found in *Lactococcus lactis* subsp. *cremoris* KW2 (Panel A) and ESX system (Panel B).

Supplementary Tables

(if only legends are given the Tables are supplied as separate Excel files)

Supplementary Table 1: OG tables including all analyzed OGs (Sheet 1), subspecies specific OGs that only occur in either ssp. *lactis* or ssp. *cremoris* (Sheet 2) and detailed lists of OGs given in the heatmaps of Figure 3 (Sheet 3), Figure 4 (Sheet 4) and Supplementary Figure 2 (Sheet 5). See Excel file.

The alignments of all OGs are deposited at zenodo.com under DOI: 10.5281/zenodo.1471674

```
https://doi.org/10.5281/zenodo.1471674
```

Supplementary Table 2: Pseudogenes occurring in more than 8 out of 43 strains (Sheet1) and an overrepresentation analysis (Sheet 2). See Excel file.

Supplementary Table 3: Glycoside hydrolase gene cluster found in *L. lactis* strains.
+ gene present; - pseudogene; no-symbol gene absent;

Glycsyhydrolase OG	GH115 OG_2205	GH1 OG_34	GH85, CBM32 OG_1824	GH13 OG_24	GH20 OG_1863	GH38 OG_1884	GH125 OG_1883	GH92 OG_1881	GH43 OG_3849	GH43 OG_871	GH11 OG_2465
**ssp. *cremoris***											
KW2	-	+	-	+	+	+	+	+		+	+
KW10	+	+	+	+	+	+	+	+		+	+
V4	+	+	+	+	+	+	+	+		+	+
N41	-	+	+	+	-	-	+	+	-	+	+
MG1363	-	-									
NCDO763	-	-									
A76	+	+									+
UC509.9	+										-
SK11	-	+									+
SK110	-	+									+
AM2	-	+									+
B40	+										-
HP	+										+
FG2	+										-
LMG6897	+										+
**ssp. *lactis***											
KF147	+	+	+	+	+	+	+	+	+	+	
M20		+	+	+	+	+	+	+		+	
KF201		+	+	+	-	+	+	+		+	
K337	+	+		+	-	+	+	+		+	
KF67		+	+	+	+	+	+	+		+	
KF134		+	+	+	+	+	+	+		+	
KF146		+	+	+	+	+	+	+		+	
KF196		+	+	+	+	+	+	+		+	
E34		+	+	+	+	+	+	+		+	
KF7		+	-	+	+	+	+	+		+	
LMG9446		+	+	+	-	+	+	+		+	
LMG8526		+	+	+	+	+	+	+	+	+	
KF282	+	+	+	+	+	+	+	+	+	+	
LMG8520		+	-	-	-	+	-	+		-	
N42		+	+	+	+	+	+	+		+	
ATCC19435		+	+	+	+	+	+	+		+	
K231		+	+	+	+	+	+	+		+	
Li-1		+	+	+	+	+	+	+		+	
IO-1		+	+	+	+	+	+	+		+	
KF24		+	+	+	+	+	+	+		+	
LMG9447		+	+	+	-	+	+	+		+	
UC317		+	+	+	+	+	+	+		+	
ML8		+	+	+	+	+	+	+		+	
CV56		+	+	+	+	+	+	+		+	
IL1403		+		+	+	+	+	+		+	
DRA4		+		+	+	+	+	+		+	
LMG14418		+		+	+	+	+	+		+	

Supplementary Table 4: Genes involved in acid stress. Symbols: +, intact gene; -, gene missing; P, pseudogene; T/+, transposase inserted adjacent to intact gene; P/T, transposase inserted adjacent to a pseudogene.

	OG	KW2	KW10	V4	N41	NCDO763	MG1363	AM2	SK11	SK110	A76	UC509.9	B40	FG2	HP	LMG6897
Genotype		*cremoris*						*cremoris*								
Phenotype		*lactis*						*cremoris*								
Arginine deiminase system																
argR	OG_1521	+	+	+	+	+	+	+	+	+	+	+	+	+	+	+
argS	OG_1099	+	+	+	+	+	+	+	+	+	+	T/+	+	+	+	+
arcA	OG_1100	+	+	+	+	+	+	P	P	P	+	+	P	P	P	P
arcB	OG_1101	+	+	+	+	+	+	+	+	+	+	+	+	+	+	+
arcD1	OG_11	+	+	+	+	+	+	+	+	+	+	+	P	P	P	P
arcC1	OG_1761	+	+	+	+	+	+	+	+	+	+	+	P	P	P	P
arcC2	OG_1522	+	+	+	+	+	+	+	+	+	+	+	+	+	+	+
arcT	OG_162	+	+	+	+	+	+	+	+	+	P/T	+	+	+	+	+
arcD2	OG_11	+	+	+	+	+	+	+	+	+	+	+	+	+	+	+
Agmatine deiminase system																
aguR	OG_1903	+	+	+	+	-	-	-	-	-	-	-	-	-	-	-
aguB	OG_1902	+	+	+	+	-	-	-	-	-	-	-	-	-	-	-
aguD	OG_1901	+	+	+	+	-	-	-	-	-	-	-	-	-	-	-
aguA	OG_1922	+	+	+	+	-	-	-	-	-	-	-	-	-	-	-
aguC	OG_1921	+	+	+	+	-	-	-	-	-	-	-	-	-	-	-
Glutamate decarboxylase system																
gadB	OG_1672	+	-	P	+	+	+	P	P	P	P	P	P	P	P	P
gadC	OG_1701	+	-	+	+	+	+	+	+	+	+	+	+	P	P	P
gadR	OG_1614	+	-	+	+	+	+	+	+	+	+	P	+	+	+	+
Malolactic system																
mleR	OG_1447	+	+	+	P	+	+	+	+	+	P	P	+	P	P	P
mles	OG_94	+	+	+	+	+	+	P	P	P	P	+	P	P	P	P
mleP	OG_944	+	+	+	+	+	+	+	+	+	P	P	+	+	+	+

Supplementary Table 5: Occurrence of genes predicted to be involved in maltose and trehalose utilization in the genomes of *L. lactis* ssp *cremoris* strains. Symbols: +, intact gene; -, gene missing; P, pseudogene; (+) intact gene present but predicted protein has only ~60% amino acid identity to that from KW2.

Function	Protein	OG	KW2	KW10	V4	N41	NCDO763	MG1363	AM2	SK11	SK110	A76	UC509.9	B40	FG2	HP	LMG6897
Genotype			*cremoris*						*cremoris*								
Phenotype			*lactis*						*cremoris*								
**Trehalose utilization**																	
Trehalose operon transcriptional repressor	GntR	OG_989	+	+	+	+	+	+	+	+	+	+	P	+	+	+	+
PTS system, trehalose-specific IIA component(EC 2.7.1.69)	PTS IIA	OG_138	+	+	+	+	+	+	+	+	+	+	P	+	P	P	P
PTS system, trehalose-specific IIB component(EC 2.7.1.69) / PTS system, trehalose-specific	PTS IIBC	OG_990	+	+	+	+	+	+	+	+	+	P	+	P	+	+	+
Trehalose 6-phosphate phosphorylase (EC2.4.1.216)	GH65	OG_1490	+	+	+	+	+	+	+	+	+	+	+	+	+	+	+
Beta-phosphoglucomutase (EC 5.4.2.6)	PGM	OG_991	+	+	+	+	+	+	+	+	+	+	+	+	+	+	+
**Maltose utilization (1)**																	
Maltose operon transcriptional repressor MalR,LacI family	LacI	OG_1364	+	+	+	+	+	+	+	+	+	+	+	+	+	+	+
Maltose phosphorylase (EC 2.4.1.8) / Trehalosephosphorylase (EC 2.4.1.64)	GH65	OG_1365	+	+	+	+	+	+	+	+	+	+	+	P	P	P	P
Alpha-glucosidase (EC 3.2.1.20)	GH13	OG_1753	+	+	+	+	+	+	+	+	+	+	P	-	-	-	-
Alpha-amylase (EC 3.2.1.1)	GH13*	OG_1768	+	+	+	+	+	+	+	+	+	+	P	-	-	-	-
Maltose O-acetyltransferase (EC 2.3.1.79)	MOAT	OG_1754	+	+	+	+	+	+	+	+	+	+	+	-	-	-	-
Glucan 1,6-alpha-glucosidase (EC 3.2.1.70)	GH13	OG_24	+	+	+	+	+	+	+	+	+	+	+	-	-	-	-
Neopullulanase (EC 3.2.1.135)	GH13	OG_1755	+	+	+	+	+	+	+	+	+	+	+	-	-	-	-
Maltose/maltodextrin ABC transporter, substratebinding periplasmic protein MalE	Maltose SBP	OG_1756	+	(+)	+	+	+	+	(+)	(+)	(+)	(+)	(+)	-	-	-	-
Maltose/maltodextrin ABC transporter, permeaseprotein MalF	Maltose P	OG_1757	+	(+)	+	+	+	+	(+)	(+)	(+)	(+)	(+)	-	-	-	-
Maltose/maltodextrin ABC transporter, permeaseprotein MalG	Maltose P	OG_1758	+	(+)	+	+	+	+	(+)	(+)	(+)	(+)	P	-	-	-	-
**Maltose utilization (2)**																	
Transcriptional regulator, LacI family	LacI	OG_3122	+	+	+	+	+	+	-	-	-	-	-	-	-	-	-
membrane protein	HP	OG_3121	+	+	+	+	+	+	-	-	-	-	-	-	-	-	-
hypothetical protein	HP	OG_3120	+	+	+	+	+	+	-	-	-	-	-	-	-	-	-
Maltose phosphorylase (EC 2.4.1.8) / Trehalosephosphorylase (EC 2.4.1.64)	GH65	OG_3119	+	+	+	+	+	+	-	-	-	-	-	-	-	-	-
Maltose/maltodextrin ABC transporter, substratebinding periplasmic protein MalE	SBP	OG_3118	+	+	+	+	+	+	-	-	-	-	-	-	-	-	-
Maltose/maltodextrin ABC transporter, permeaseprotein MalF	P	OG_3093	+	+	+	+	+	+	-	-	-	-	-	-	-	-	-
Maltose/maltodextrin ABC transporter, permeaseprotein MalG	P	OG_2956	+	+	+	+	+	+	-	-	-	-	-	-	-	-	-

* Gene has a signal peptide sequence and is predicted to encode a secreted enzyme.

Supplementary Table 6: Chromosomal genes linked to citrate metabolism found in sequenced strains. Genes from IL1403, DRA4 and M20 are homologous and show >99% amino acid identity. The genes from KF67 show only 54-79% amino acid identity relative to IL1403 and presumably have a different origin.

<table>
<tr><th>Gene</th><th>OG</th><th>IL1403</th><th>DRA4</th><th>M20</th><th>KF67</th></tr>
<tr><td>Acetolactate synthase*</td><td>OG_753</td><td>+</td><td>+</td><td>+</td><td>+</td></tr>
<tr><td>CitM</td><td>OG_3414</td><td>+</td><td>+</td><td>+</td><td>+</td></tr>
<tr><td>Malate permease (CitP)</td><td>OG_2951</td><td>P</td><td>P</td><td>+</td><td>+</td></tr>
<tr><td>CitR</td><td>OG_3413</td><td>+</td><td>+</td><td>+</td><td>+</td></tr>
<tr><td>CitC</td><td>OG_3412</td><td>+</td><td>+</td><td>+</td><td>+</td></tr>
<tr><td>CitD</td><td>OG_3411</td><td>+</td><td>+</td><td>+</td><td>+</td></tr>
<tr><td>CitE</td><td>OG_3410</td><td>+</td><td>+</td><td>+</td><td>+</td></tr>
<tr><td>CitF</td><td>OG_3409</td><td>+</td><td>+</td><td>+</td><td>+</td></tr>
<tr><td>CitX</td><td>OG_3572</td><td>P</td><td>+</td><td>+</td><td rowspan="2">Fused CitXG</td></tr>
<tr><td>CitG</td><td>OG_3408</td><td>-</td><td>P</td><td>+</td></tr>
</table>

*The citrate utilization genes are immediately downstream from acetolactate synthase (*als*) in IL1403 and DRA4. In M20 the *als* and citrate metabolism genes are on separate small contigs. In KF67 the citrate metabolism genes are not adjacent to *als* and the *cit*X and *cit*G genes are fused.

Supplementary Table 7: NRPS-PKS clusters. Green indicates gene presence, white indicates gene absence and light blue indicates pseudogenes.

		Genotype	*cremoris*									*cremoris*						*lactis*																												
		Phenotype	*cremoris*									*lactis*						*lactis*																												
OG	gene	Annotation	FG2	HP	LMG6897	B40	UC509_9	AM2	SK110	SK11	A76	KW10	KW2	N41	V4	NCDO763	MG1363	P7266	ATCC19435	E34	KF134	KF146	KF196	KF201	KF282	K231	KF24	K337	KF67	KF7	Li1	LMG14418	LMG8520	LMG8526	LMG9446	LMG9447	M20	ML8	N42	DRA4	UC317	KLDS	IL1403	IO_1	CV56	KF147
OG_5219		hypothetical protein																																												
OG_3857	npkK	FIG00525014: hypothetical protein																																												
OG_3858	npkR	Two-component response regulator yvqC																																												
OG_3859	nrpA	Peptide synthetase																																												
OG_3860	pksA	Malonyl CoA-acyl carrier protein transacylase(EC 2.3.1.39)																																												
OG_3081	pksB	Malonyl CoA-acyl carrier protein transacylase(EC 2.3.1.39)																																												
OG_3081	pksC	Malonyl CoA-acyl carrier protein transacylase(EC 2.3.1.39)																																												
OG_3524		ABC transporter, ATP-binding protein																																												
OG_3525		ABC transporter, permease protein																																												
OG_3861		hypothetical protein																																												
OG_3526	npkS	Malonyl CoA-acyl carrier protein transacylase(EC 2.3.1.39)																																												
OG_3862	nrpB	Thioesterase																																												
OG_3863	nrpC	4'-phosphopantetheinyl transferase (EC2.7.8.-)																																												
OG_3864		hypothetical protein																																												
OG_3646	avrD	Avirulence AvrD-like protein																																												
OG_3865		putative FAD-dependent oxidoreductase																																												
OG_3645		putative ATP/GTP-binding protein, MmyX																																												
OG_5218		hypothetical protein																																												
OG_3866		hypothetical protein																																												
OG_3867		hypothetical protein																																												
OG_2651		Phage integrase																																												
OG_3868		hypothetical protein																																												
OG_3681		hypothetical protein																																												
OG_2132		Mobile element protein																																												
OG_3869		protein of unknown function DUF323																																												
OG_3870		amino acid adenylation domain																																												
OG_2230		amino acid adenylation domain																																												
OG_2099		Collagen binding protein Cna																																												
OG_2125		Osmosensitive K+ channel histidine kinase KdpD(EC 2.7.3.-)																																												
OG_2124		sensor histidine kinase																																												
OG_2123		Potassium-transporting ATPase A chain (EC3.6.3.12) (TC 3.A.3.7.1)																																												
OG_2122		Potassium-transporting ATPase B chain (EC3.6.3.12) (TC 3.A.3.7.1)																																												
OG_2098		Potassium-transporting ATPase C chain (EC3.6.3.12) (TC 3.A.3.7.1)																																												

Supplementary Table 8: Details of surface polysaccharide gene clusters. Each worksheet presents different sets of strains compared to reference strain KF147. Color codes: Green, putative plasmid contigs; grey, genes bordering polysaccharide gene clusters; light blue, pseudogenes. See Excel file.

Supplementary Table 9: Grouping of strains with a similar RGP cluster (rhamnose-glucose polysaccharide)

Group 1	DRA4	LMG852	KLDS	N42	LMG144		
Group 2	ATCC19	K231	KF24	Li1	LMG944		
Group 3	KF7	IL1403	KF147				
Group 4	KF196	KF134	KF146	LMG944			
Group 5	KF67	ML8	UC317	CV56	UC509_9		
Group 6	LMG852	E34					
Group 7	FG2	HP	LMG689	NCDO76		MG1363	
Group 8	B40	N41				KW2	
Group 9	SK110	SK11	AM2	KF282			
Ungroupable	A76	V4	IO_1	K337	KW10	KF201	M2

Supplementary Table 10: Prophage insertion location (see details on numbering below the table). Integration loci 1-7 where described earlier (Kelly et al 2013) while loci 8-12 were identified here.

	Prophage insertion location											
**Strain**	1	2	3	4	5	6	7	8	9	10	11	12
KW10												
KW2					X							
V4		X	X	X	X				X			
N41						X						
NCDO763		X				X						
MG1363		X				X						
SK110						X						
AM2				X		X						
SK11		X		X		X						
A76	X				X	X						
UC509.9	X					X						
B40		X	X		X	X						
FG2				X		X						
HP				X		X						
LMG6897				X		X						
LMG8526										X	X	
KF282				X	X		X				X	
LMG8520	X											
IO-1		X		X								
K231			X		X				X			
Li-1		X										
KF24					X		X		X			X
LMG9449				X		X						
ATCC19435	X		X	X		X						
E34					X							
K337									X			
KF201		X	X									
M20		X							X			
N42	X		X				X					
DRA4	X							X				
IL1403	X		X	X								
CV56				X		X	X					
ML8			X	X		X	X					
UC317						X	X			X		
LMG14418	X					X		X				
KLDS						X	X	X			X	
KF147			X	X								
KF7					X							
KF67						X	X		X			
KF196						X						
KF146						X						X
KF134						X			X			
LMG9446		X			X							

Insertion location numbering:

1 between *lyt*R (COG1316 and *tru*A (COG0101);
2 between *rbs*B (COG1879) and *uxu*B (COG0246);
3 between *car*B (COG0458) and *coa*A (COG1972);
4 between *rex* (COG2344) and *rad*C (COG2003);
5 between *fab*K (COG2070) and *suf*B (COG0719);
6 between *sun*L (COG0144) and *fmt* (COG0223);
7 between *com*GB (COG1459) and *com*GC (COG4537);
8 between *deo*D (COG0813) and *fhs* (COG2759);
9 between *rpl*S (COG0335) and *yij*G (COG1215);
10 between *adk* (COG0563) and *rps*M (COG0099);
11 between *car*A (COG0505) and *rpm*E (COG0254);
12 between *lig*A (COG0272) and *ptc*C (COG1455);

Supplementary Table 11: Sex factor gene clusters. The four worksheets represent different sets of strains with similar sex factor gene clusters. The putative sex factor gene cluster is indicated as SexF in the column “cluster”. See Excel file.

Supplementary Data in online repository

The following data was deposited at zenodo.com under the DOI: 10.5281/zenodo.1471674

```
https://doi.org/10.5281/zenodo.1471674
```

FILE NAME	FILE DESCRIPTION
**alignments.zip**	This .zip file contains the individual fasta files and the alignmens from all OGs used in this study.
**GTM_output.zip**	This .zip file contains all output files from the genotype-phenotype matching done with the phenotypes described by Bayjanov *et al*.
